# Individual *Apostichopus japonicus* fecal microbiome reveals a link with polyhydroxybutyrate producers in host growth gaps

**DOI:** 10.1038/srep21631

**Published:** 2016-02-24

**Authors:** Yohei Yamazaki, Pedro Milet Meirelles, Sayaka Mino, Wataru Suda, Kenshiro Oshima, Masahira Hattori, Fabiano L. Thompson, Yuichi Sakai, Toko Sawabe, Tomoo Sawabe

**Affiliations:** 1 Laboratory of Microbiology, Faculty of Fisheries Sciences, Hokkaido University, Hakodate, Japan; 2 Institute of Biology, SAGE-COPPE, Federal University of Rio de Janeiro (UFRJ), Rio de Janeiro, Brazil; 3 Laboratory of Metagenomics, Graduate School of Frontier Sciences, University of Tokyo, Kashiwa, Japan; 4 Department of Microbiology and Immunology, Keio University School of Medicine, Tokyo, Japan; 5 Graduate School of Advanced Science and Engineering, Waseda University, Tokyo, Japan; 6 Hakodate Fisheries Research, Hokkaido Research Organization, Local Independent Administrative Agency, Hakodate, Japan; 7 Department of Food and Nutrition, Hakodate Junior College, Hakodate, Japan

## Abstract

Gut microbiome shapes various aspects of a host’s physiology, but these functions in aquatic animal hosts have yet to be fully investigated. The sea cucumber *Apostichopus japonicus* Selenka is one such example. The large growth gap in their body size has delayed the development of intensive aquaculture, nevertheless the species is in urgent need of conservation. To understand possible contributions of the gut microbiome to its host’s growth, individual fecal microbiome comparisons were performed. High-throughput 16S rRNA sequencing revealed significantly different microbiota in larger and smaller individuals; *Rhodobacterales* in particular was the most significantly abundant bacterial group in the larger specimens. Further shotgun metagenome of representative samples revealed a significant abundance of microbiome retaining polyhydroxybutyrate (PHB) metabolism genes in the largest individual. The PHB metabolism reads were potentially derived from *Rhodobacterales*. These results imply a possible link between microbial PHB producers and potential growth promotion in Deuterostomia marine invertebrates.

*Apostichopus japonicus* (Selenka) is a temperate sea cucumber species occurring in the western Pacific Ocean, Yellow Sea, Sea of Japan, and Sea of Okhotsk^1^. It ingests organic matter, bacteria, protozoa, diatoms as well as algae and animal detritus and thus plays an important role in benthic nutrients recycling^2^. *A. japonicus* is also an important fishery resource in many Asian countries, especially in China^3^. Wild stocks have drastically declined due to overfishing, and in 2013 this species was listed as “Endangered” on the IUCN Red List of Threatened Species^4^. Wild stocks of sea cucumber species have been sustained by seed production and aquaculture^3^, however a lack of information concerning ecology, physiology, and biochemistry of this species is an obstacle to further success in this field. Extremely large growth gap among the juveniles of *A. japonicus* (Fig. 1a) is also a common problem in the seed production of sea cucumbers^5^,^6^,^7^. As a result, there is a right-skewed body size distribution among cultured animals even when raised in the same tank under identical conditions (e.g. temperature, light, animal density). This can be seen in Fig. 1a,b in which the largest individual is ca. 50 times larger than the smallest one. Population densities, congenital hosts’ traits and food competition have all been considered as possible causes, however, the cause of this huge growth gap is still unknown^5^.

The increasing demand for *A. japonicus* livestock has led to further research seeking optimal conditions for seeding and farming. Most of the research has focused on i) the effects of abiotic factors on sea cucumbers growth^8^,^9^; ii) the effects of different diets and ration composition on growth^10^,^11^; iii) the use of stable isotopes to assess carbon turnover, metabolism, growth and absorption of different food sources^12^,^13^; iv) the effects of potential probiotics on growth, immunity and resistance to pathogens^14^,^15^; and v) the effect of stock density on growth rates^16^. Holothurians are also used as model animals to study visceral regeneration of digestive systems^17^,^18^. Despite its importance, a few studies have assessed *A. japonicus* microbial diversity using either culture-dependent or independent methods^19^,^20^,^21^.

The relationship between gut microbiota and hosts has been widely studied in many organisms. For instance, microbiome in ruminants and termites are largely responsible for the digestion of indigestible components such as cellulose and hemicellulose^22^,^23^ and is also responsible for fatty acid absorption in zebra fish^24^. Obesity has been associated with mouse gut microbiota^25^; colonization of germ-free mice with an ‘obese microbiota’ results in a significant increase in total body fat compared to colonization with a ‘lean microbiota’^26^. Gut microbiota can improve immune system response^27^ and can also modulate brain development and behavior^28^,^29^. These studies indicate a strong interaction between microbiota and host, however, little is known about that of *A. japonicus* sea cucumber, which is one of the phylogenetically close Deuterostomia species^30^.

We have observed a growth gap in juvenile sea cucumbers in Hokkaido hatcheries. Most previous studies have assessed the influence of abiotic and biotic factors on sea cucumber growth manipulating the concentrations, composition or intensity of the environmental factors^8^,^9^,^10^,^11^,^13^,^14^,^15^,^16^. However, to our knowledge, there have been no studies investigating the possible intrinsic causes of sea cucumber growth. Our hypothesis is that gut microbes play a major role on sea cucumber growth. To explore any possible contributions of the gut microbiome to its host’s growth, individual fecal microbiome comparisons (both taxonomically and functionally) of both large and small individuals of sea cucumber *A. japonicus* were performed. To reach our goal, we developed a non-destructive (without animals sacrifice) protocol for sea cucumber feces collection and DNA extraction to assess the gut microbial diversity in live specimens. We analyzed the microbial diversity taxonomically by pyrosequencing the V1-V2 region of 16S rRNA gene and functionally by massively parallel sequencing metagenomes. The non-destructive individual analysis we introduce here opens the way to both marine animal conservation and also to studies in the dynamics of host-microbes interaction.

## Results

### Larger and smaller *Apostichopus japonicus* have different fecal microbial communities

We assessed fecal microbiota of the larger and smaller individual sea cucumbers using tag sequencing of the V1-V2 region of 16S rRNA gene. After trimming, 104,679 qualified reads from the sea cucumber samples (52,996 and 51,683 reads from 10 larger and smaller individual samples, respectively) and 5,241 qualified reads from the seawater sample were used to cluster into OTUs (similarity 97%) (Table S1). After removing *Eukarya*, mitochondria and chloroplast sequences, we used 63,574 bacterial sequences for taxonomic assignment. Chloroplast sequences, which might be from ingested algae, were more frequently associated with smaller individuals [48.4 ± 16% (mean ± SD)] than larger individuals (37 ± 15%), however, these were not significant (*P* > 0.05). In total 1,976 OTUs with average Good’s coverage of 93.5 ± 1.8% were obtained. Eukaryotic diversity, consisting of >90% of stramenopiles, was not significantly different between the two groups.

The fecal microbiota of the larger and the smaller individuals were clustered separately from one another, and separated from the seawater microbiota from the rearing tank (Fig. 2a). We found two major taxa in the fecal microbiota. The most abundant phylum was *Proteobacteria*; 57.4 ± 4.4% and 52.7 ± 4.9% of reads in the larger and the smaller individuals, respectively. *Proteobacteria* accounted for 77.3% of reads in the seawater sample. *Bacteroidetes* was the second most abundant phylum in sea cucumber samples (34.0 ± 4.8% and 37.8 ± 6.3% in larger and smaller individuals, respectively). Only 11.4% of this phylum was observed in the seawater sample. Comparing classes among *Proteobacteria* in the fecal microbiota, the relative abundances of *Alphaproteobacteria* (Larger, 15.3 ± 1.8%; Smaller, 11.5 ± 2.8%) and *Deltaproteobacteria* (Larger, 2.3 ± 0.7%; Smaller, 1.1 ± 0.8%) were significantly different between the larger and smaller individuals (*P* < 0.05, *q* < 0.05). The relative abundances of minor phyla (*Actinobacteria*, *Firmicutes*, *Fusobacteria* and *Spirochaetes*) were significantly different between the two groups (*P* < 0.05, *q* < 0.05, see Table S2).

The most abundant order was *Flavobacteriales* (Larger, 29.0% ± 5.2%; Smaller, 33.5% ± 6.4%), followed by *Alteromonadales* (Larger, 23.9% ± 5.0%; Smaller, 25.1% ± 3.2%), but the data was not significantly different between the two groups (*P* > 0.05). *Rhodobacterales* among *Alphaproteobacteria* was the third most abundant (Larger, 14.8 ± 2.0%, and Smaller, 11.1 ± 2.8%), and the relative abundances between larger and smaller individuals were significantly different (Fig. 2b and Table S2 (*P* < 0.05, *q* < 0.05)). *Desulfobacterales* (Larger, 1.1% ± 0.5%; Smaller, 0.2% ± 0.1%) (*Deltaproteobacteria*) and *Oceanospirillales* (Larger, 0.9% ± 0.7%; Smaller, 0.3% ± 0.4%), were more abundant in larger individuals (*P* < 0.05, *q* < 0.05), on the other hand *Marinicellales* (Larger, 2.8% ± 0.6%; Smaller, 3.5% ± 0.7%) and *Acidimicrobiales* (Larger, 0.9% ± 0.3%; Smaller, 1.3% ± 0.4%) were more abundant in smaller individuals (*P* < 0.05, *q* < 0.05). Relative abundances of some other orders, for instance *Fusobacterales*, *Rhizobiales* and *Actinomycetales*, were also significantly different between the two groups (*P* < 0.05, *q* < 0.05), but these were quite low occupation (<1%) (Fig. 2b).

Species richness and diversity were not statistically significant between the two groups of microbiota (Fig. 3a–c), on the contrary, the unweighted UniFrac analysis demonstrated clear clustering of the larger and smaller individuals microbiota (Fig. 3d).

### Core fecal microbiota of *Apostichopus japonicus* juveniles and OTUs correlated to hosts’ body weight

We hypothesized that larger and smaller sea cucumbers could possess unique microbiota. Comparisons of individual fecal microbiota of *A. japonicus* demonstrated that 30, 12 and 35 OTUs were defined as larger individual core (LIC), smaller individual core (SIC), and all individual core (AIC) microbiota, respectively (Fig. S1a). The OTUs from LIC and SIC microbiota were further affiliated to 10 and 6 major bacterial orders, respectively. *Rhodobacterales*, *Alteromonadales*, *Cytophagales*, HTCC2188 (*Gammaproteobacteria*) and *Myxococcales* were absent in the SIC, and *Marinicellales* was not observed in the LIC microbiota (Fig. S1b). Those of the AIC microbiota were classified into nine orders (e.g. *Rhodobacterales*, *Flavobacteriales*, and *Alteromonadales*). A total of 1,728 OTUs were also defined as a pan fecal microbiota of the cultured juvenile *A. japonicus*.

We further identified that 15 OTUs had strong positive correlation with sea cucumber body weight (*r* > 0.7) with statistical significance (corrected *P* < 0.05), respectively (Table S3). Two and six OTUs were affiliated to LIC and larger only, respectively. OTU584 (*Legionellaceae*) showed the highest positive correlation. OTU1150 (*Alteromonadales* OM60) was found both in all *A. japonicus* individuals used in this study and the seawater.

### Comparative metagenome analysis of fecal microbiome

Metagenome sequencing using the HiSeq platform was performed on the largest and the smallest specimens (except specimens containing insufficient fecal DNA samples) as representatives of larger and smaller individuals used in the above analyses. From the largest and the smallest individuals, 22,386,506 reads (2.2 Gb) and 22,803,396 reads (2.3 Gb) were obtained, respectively. After quality filtering, 18,582,516 and 18,156,817 reads from the largest and the smallest individual, respectively, were used for MG-RAST annotation. The total number of annotated reads was 6,049,986 and 4,634,631 for the largest and the smallest individual, respectively. Bacterial reads occupied 92.6% and 87.6%, eukaryotic reads occupied 7.2% and 12.2%, and archaeal reads occupied 0.16% and 0.15% in the largest and the smallest library, respectively. Bacterial community based on the metagenomic reads was similar to those obtained by the 16S rRNA gene based microbiota analysis (Fig. S4).

The metagenomic analysis demonstrated that 25 functional features on subsystem category (Level 1) were significantly more abundant in one of the samples. Microbial reads annotated to ‘carbohydrates’, ‘amino acids and derivatives’, ‘cofactors, vitamins, prosthetic groups, pigments’, ‘fatty acids, lipids, and isoprenoids’, ‘cell wall and capsule’ and others were significantly more abundant in the largest individual, and ‘protein metabolism’, ‘RNA metabolism’, ‘respiration’, ‘virulence, disease and defense’ and others were significantly more abundant in the smallest individual (Fig. S2a). In more detail, the reads affiliated to genes responsible for polyhydroxybutyrate (PHB) metabolism, n-phenylalkanoic acid degradation, fatty acid metabolism cluster, acetyl-CoA fermentation to butyrate, fatty acid degradation regulons, serine-glyoxylate cycle and butyrate metabolism in Level 3 subsystem category were significantly more abundant in the largest individual. Among those, the largest positive effect size (more abundant in the largest individual) was observed in reads affiliated to PHB metabolism (Fig. 4). Interestingly further KEGG mapping of the annotated reads into the PHB metabolism to validate which metabolic pathways are more prominent revealed *phaA* (acetyl-CoA C-acetyltransferase), *phaB* (acetoacetyl-CoA reductase) and *phaC* (PHB synthase), which are essential in PHB synthesis from acetyl-CoA to PHB, were more abundant in the largest individual’s metagenomic library (Fig. S3a). In particular, the abundance of *phaA* gene (EC 2.3.1.9) reads impacted not only mapping into PHB metabolism but also that of other subsystem categories (Fig. S3b–g). The abundance of enoyl-CoA hydratase (EC 4.2.1.17), which is involved in butyrate metabolism, lysine and tryptophan synthesis and PHB degradation, also affected to positive effective size of most of the subsystem categories (Fig. S3a–e,g). Notably, 67% in average of total prokaryotic reads annotated to the *phaABC* genes were affiliated to *Rhodobacterales*, in accordance with the results obtained by 16S rRNA typing, where higher abundance of *Rhodobacterales* in the larger individuals’ microbiota was found.

Reads affiliated to essential cellular functions in subsystem Level 3 categories (e.g. bacterial ribosome LSU or SSU, bacterial RNA polymerase, F0F1-type ATPase) were significantly more abundant in the metagenomic library from the smallest animal (Fig. S2b). Validation of the taxonomic affiliation showed, for example, bacterial RNA polymerase reads were consisted of diverse array of the sea cucumber gut microbiome.

## Discussion

*A. japonicus* sea cucumber is a good candidate to study the evolution, dynamics and functions of gut microbiome in Deuterostomia invertebrates due to their unique ecophysiology^17^,^18^, and it is in urgent need of both conservation and development of intensive aquaculture^3^,^4^. To our knowledge, this is the first individual fecal microbiome analysis of *A. japonicus* to assess the possible interactions between the microbiome and the host animals’ growth. Our analyses showed that the observed differences in fecal microbiota of larger and smaller individuals might be associated with PHB synthetic metabolism. Interestingly, both high-throughput sequencing approaches suggest that *Rhodobacterales* may play a key role in sea cucumber growth. Feeding trials assessing direct effect and causality of these gut microorganisms on their hosts’ growth are necessary to support this idea, but our results provide a new insight into a link between PHB producing microbes and their potential contribution to sea cucumber growth. Only a few studies have investigated the gut microbiota of holothurians sea cucumbers, all of them using dissection techniques. These studies have assessed gut bacterial diversity and concluded selective feeding and proposed some potential probiotic bacteria^19^,^31^. None of these previous studies have investigated the roles of bacterial communities on the host’s health or growth. Using the non-destructive methodology presented here, live specimens can be used to monitor gut microbial dynamics and host-microbes interaction; for example, the dynamics of gut microbiome before and after evisceration, and after probiotic treatment could be monitored. Again, we would like to emphasize that sea cucumber is one of the best model animals to study in terms of organ regeneration among Deuterostome animals in both molecular and genetic basis. In fact, studies have revealed numbers of novel genes differentially expressed during the gut regeneration process^17^. It is interesting to understand how the gut microbiome, including PHB producers, is affected such examples of the regeneration process using the non-destructive fecal microbiome methodology. The strategy also aids to the “endangered” species conservation in the wild. Without unnecessary mortality, on site fecal collections of a Stichopodidae sea cucumber and further analyses are in progress.

We found a similar number of bacterial OTUs and diversity (Shannon index) in *A. japonicus* gut microbiota to other studies using similar DNA extraction and pyrosequencing strategies^19^. The diversity indices of the *A. japonicus* fecal microbiota are similar to those observed in *Hydra* microbiota but lower than those of fish and mammals^32^. The fecal microbiota of juvenile *A. japonicus* is predominantly in *Proteobacteria* and *Bacteroidetes*, whereas *Bacteroidetes* was less dominant in maricultured adult specimens^19^. It is well known that gut microbiota is influenced by food intake, by environmental factors and by developmental stages of hosts and their genetics^32^,^33^. The observed differences in microbiota along with sea cucumber’s body weight may be caused by one of (or synergy among) the above-mentioned factors. Similar to the previous report on host habitat selection of the gut microbiota of zebrafish and mouse^34^, 8 and 10 phyla detected in the sea cucumber microbiota were shared with those of mice/human and zebrafish, however the abundance and diversity below phylum level differed. The juvenile *A. japonicus* individuals also showed microbiota signatures observed in both fish guts (*Proteobacteria* rich) and the human gut (*Bacteroidetes* rich)^32^. In the previous report on gut microbiota transplantation experiments between zebrafish and mouse, a restoration tendency to the original composition of gut microbiota is observed; abundance of *Proteobacteria* increases, but that of *Bacteroidetes* drops to below the detectable limit in a mouse-microbiota transplanted zebrafish^34^. A hybrid type of sea cucumber microbiota observed here might be affected by not only their conserved status of these hosts in surviving in aquatic environments but also their host species specific selective pressure. Archaeal sequences were found to be rare in the metagenomic libraries. Similar results were found in a previous study^35^, therefore it is not necessary to discuss their roles in the gut microbiota here.

*Rhodobacterales*, *Desulfobacterales*, and *Oceanospirillales* (in more detail *Rhodobacteraceae* and *Desulfobulbaceae*; see family table in TableS2) were significantly abundant in the larger individuals, and *Marinicellaes* and *Acidimicrobiales* were similarly abundant in the smaller. *Rhodobacteraceae* is one of the most diverse aquatic bacterial groups (>100 genera) consisting of aerobic photo- and chemoheterotrophs and a diverse array of polyhydroxyalkanoates (PHA), commonly PHB, producers isolated from marine environments^36^,^37^,^38^. *Rhodobacteraceae* sequences and isolates have frequently been observed in marine environments^37^ as well as in the gut microbiota of wild and maricultured *A. japonicus*^19^,^35^. More recently, dietary β-glucan supplementation for *A. japonicus* triggered *Rhodobacteraceae* abundance in the gut microbiota and host NF-κB signaling pathway, and then it was speculated that the activation of the signaling pathway was caused by the increased mass of the bacterial group^20^. *Rhodobacteraceae* can be involved in sulfur and carbon biogeochemical cycling in water columns and on the photic sea floor due to their physiological traits. This bacterial group is being considered as transient fractions in the gut microbiota from the environments^35^. Interestingly, the metagenomic comparisons described here reveal a further possible hypothesis that *Rhodobacterales* affects the host growth of *A. japonicus* as a PHB producer. PHB is known to be accumulated in commonly nutrient-limited bacterial cells, especially under increased carbon to nitrogen ratio conditions^39^,^40^. There are three types of PHB, 1) high molecular weight storage PHB consisting of >1,000 3-hydroxybutyrate (3HB) residues, 2) low molecular weight PHB which has ca. 100 to 200 3HB units and 3) conjugated PHB (cPHB) consisting of low numbers of 3HB units (<ca. 30) which are covalently linked to proteins^40^. PHB-like granules have recently been observed in human cells^41^. PHB degradation is also catalyzed by PHB/PHA depolymerase in the first step of biodegradation, and the degradation process can serve as energy sources for the host cells. PHB has been tested as feed additives promoting host growth in fish and crustacean aquaculture^42^,^43^,^44^. The PHB also conferred protection to an *Artemia* host against a pathogenic vibrio by modulating the Hsp70-triggered innate immune response^45^. A PHB depolymerase gene (*phaZ*) was also retrieved from an EST library from the intestinal tissues of *A. japonicus* (GenBank JK730361.1). It is possible to consider that the bacterial PHB, especially that produced by *Rhodobacterales*, might also serve as an energy source for a host sea cucumber, causing the observed higher growth in the larger individuals. PHB producing bacteria has also been found in termite guts, but its proper role has not been fully elucidated yet^46^,^47^. This hypothesis further gives us a novel ecological insight into carbon cycling as a bridge between storage granule producing bacteria and extra-nutrition for benthic marine invertebrates. Considering our results, we suggest the use of PHB producers as probiotics in sea cucumber aquaculture. Future bioassays studies using isolated PHB producing bacteria or their PHB are necessary to confirm their probiotics potential in promoting sea cucumber growth.

Another group of bacteria more abundant in larger individuals was *Desulfobulbaceae*, which is a strictly anaerobic sulfate-reducing bacteria (SRB)^48^,^49^. The presence of *Desulfobulbaceae* sequences suggests that anoxygenic environments are formed in the gut of *A. japonicus* juveniles. In a previous study, *Desulfosarcina*, a member of *Desulfobacterales*, was also predominant in the hindgut contents of the maricultured *A. japonicus*^19^. The roles of SRB are presumed to be related to acetate production and nitrogen fixation^19^. SRB colonizes ca. 50% of human guts, and are frequently detected in healthy adults fecal microbiota^50^. SRB are known to be the most efficient hydrogenotrophs, they can utilize H_2_ and some fermentation products (e.g. lactate, formate) produced in the guts as electron donors^49^. Thus, SRB contribute towards maintaining redox balance and maximizing microbial energy production. The higher abundance of SRB sequences observed in larger individuals’ feces suggests higher redox states in their gut. Such conditions are more likely to be suitable for fermentation and PHB accumulation, which might affect the host’s growth.

The lack of information on detection and/or isolation of *Oceanospirillales*, *Marinicellales*, and *Acidimicrobiales* in the growth of the host sea cucumber resulted the delays in discovering details in the structure-function relationships. However, *Halomonas meridiana* and *Marinomonas pontica* (*Oceanospirillales*) have been isolated from sea sediment of the host ’s habitat^21^, and the guts of *A. japonicus*^35^. In particular, the *Halomonas* strain showed polysaccharide degrading abilities (starch and carboxymethyl cellulose sodium salt)^21^, which expects the roles in supporting roles in digestion of ingested detritus by the host animals. *Marinicellales* reads were detected from hind gut of *Apostichopus japonicus* using 16S rRNA gene sequencing analysis^19^, and *Acidimicrobiales* strains were isolated from the abdominal epidermis of a sea cucumber *Holothuria edulis*^51^. Both groups of bacteria actually might have associations with the sea cucumber, further ecophysiological and ecogenomics studies could infer the host effects on the unique host animals.

We found a strong positive correlation between OTUs affiliated to *Legionellaceae* and *Alteromonadales* OM60 to the body weight of *A. japonicus* juveniles. *Legionella* spp. are related to PHB production^52^, and *Alteromonadales* OM60 (such as *Congregibacter* spp.^53^) is related to polyphosphate (polyP) production. Recently, in mammalian cells, cPHB has been found in a wide variety of tissues and in atherosclerotic plaques, and polyP has been linked to a variety of functions, including blood coagulation, cell proliferation, apoptosis and mitochondrial ion transport and energy metabolism^54^. Those bacterial polymers might trigger unique biological functions in host Deuterostomia invertebrates. Once the specific functions are unveiled, these bacteria could also be candidates for probiotics in sea cucumber aquaculture.

In conclusion, we firstly assessed possible links between gut microbiome and host growth in cultured *A. japonicus* juveniles. We suggest that *Rhodobacterales* bacteria retaining PHB metabolism genes might contribute to the production of larger individuals. Our data also revealed a healthy microbiome in larger individuals composed of SRB, commonly observed in human healthy adults, in balancing the redox state of gut environments. These results imply a link between microbial PHB producers and potential growth promotion in marine invertebrates. We need further studies using gut microbiota transplant from large to small sea cucumber gnotobiotic individuals to assess the direct effects of gut microbes on sea cucumbers’ growth.

## Methods

### Sea cucumbers sampling

Cultured sea cucumber *A. japonicus* was used for this study. The animals were fertilized and grown in Hokkaido Fisheries Station, Muroran, Japan. The juveniles were fed with cultured diatoms and an artificial diet (Norwegian *Ascophyllum nodsum* powder) for 18 months. These animals were then moved to a farm in Kumaishi, Japan, in June 2014, and then were acclimated with naturally occurring diatoms for two months in a 500 ton tank and reared in sand-filtered coastal seawater pumped up from 10 m depth. Seawater temperature in the tank ranged from 8.5 °C to 13 °C.

**Figure 1:**
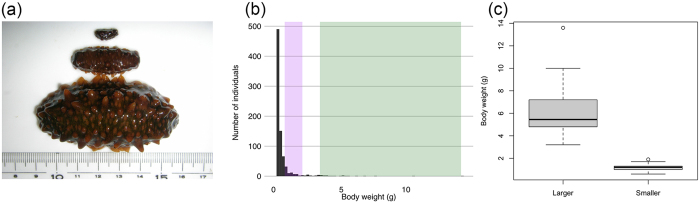
Growth gaps observed in the cultured sea cucumbers. (**a**) Representatives of the larger and the smaller sea cucumbers cultured under identical conditions are displayed from lower to upper. (**b**) A histogram for body weight of sea cucumbers showed right-skewed size distribution. These specimens are 20 months old, and were raised on artificial diets for 17 months. For the final two months in the Kumaishi farm, the animals were fed with naturally occurred diatoms before being studied. Size distributions of the larger and smaller individuals used in this study were highlighted in green and purple, respectively. (**c**) Box plot for actual body weights from 10 larger and 10 smaller individuals used in this study. The body weight averages of two groups, “larger” and “smaller”, were statistically significant.

**Figure 2:**
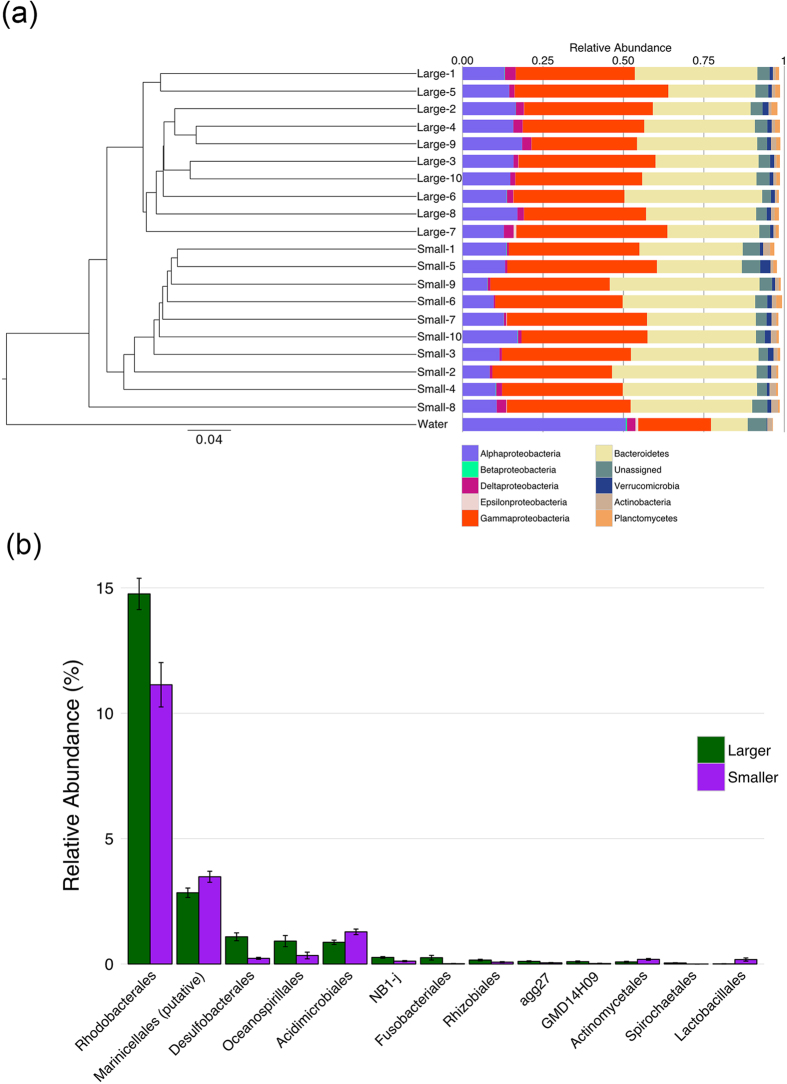
The microbiota of larger and smaller sea cucumbers feces are different. (**a**) The larger and the smaller sea cucumbers clustered individually, and seawater sample differed from sea cucumber samples. (**b**) Ranking of the order level abundances in fecal microbiota of the larger (green) and the smaller (purple) individuals. Only significant taxa were shown in this bar plot; *Rhodobacterales*, *Desulfobacterales* and *Oceanospirillales* were significantly more abundant in the larger individuals, *Marinicellales*, *Acidimicrobiales* were more abundant in the smaller individuals.

**Figure 3:**
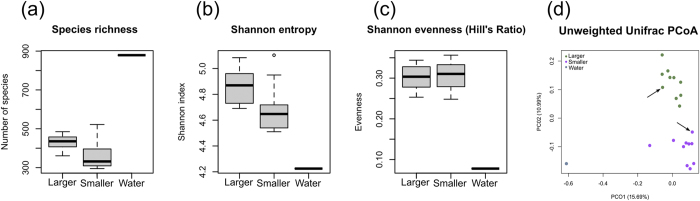
Microbial diversity comparisons between larger and smaller *Apostichopus japonicus* individuals. (**a**) Species richness, (**b**) Shannon index, (**c**) evenness, and (**d**) unweighted UniFrac-based 2D PCoA plot was based on all OTUs from the larger, the smaller sea cucumbers and seawater sample. Percent variation expected were PC1 15.69%, PC2 10.99%. Samples indicated with arrows were used for functional metagenomic analysis. The larger sea cucumbers, the smaller ones, and seawater sample are indicated in green, purple, and blue, respectively.

**Figure 4:**
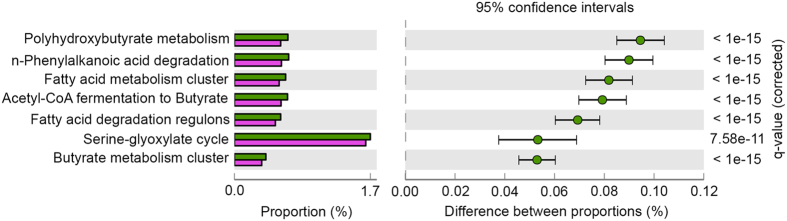
An extended error bar plot showing more abundant features in the largest sea cucumber. Green bars indicate the largest sea cucumber and purple indicate the smallest sea cucumber. Proportion (left side) means a possible abundance of microbes possessing each functional feature, and difference between proportions (=effect sizes) for each feature is indicated by a green dot. For this analysis, features were filtered by *q* value (0.05) and effect size (0.05). Relative abundances of metagenome reads related to polyhydroxybutyrate (PHB) metabolism and butyrate metabolism were more abundant in the largest individual.

In order to verify the sea cucumber body weight distribution, 816 individuals from one tank were weighed (Fig. 1b). The mean body weight and the median were 0.41 g and 0.14 g, respectively. We categorized two groups of these individuals from the top 20% because the smaller individuals within this criterion were too small for feces sampling. We classified large individuals as weighing >2.0 g as ‘larger sea cucumbers’ and small individuals as weighing <1.9 g as ‘smaller sea cucumbers’.

### Collection of individual sea cucumber feces and seawater

For bacterial DNA preparation, we collected feces of individual sea cucumber to avoid sacrificing animals and seawater in a rearing tank under semi-aseptic conditions on site. All sampling procedures were performed inside an instant clean booth, illuminated by ultraviolet light for 15 minutes (GL-15, Panasonic, Osaka, Japan). For feces collection, we selected 10 individuals from each group (larger and smaller) (Fig. 1b,c). Sea cucumber individuals were cleaned using filter-sterilized seawater and moved individually into sterile beakers with 300 mL of filter-sterilized seawater until feces was released (~20 min). The filter-sterilized seawater was prepared as follows; natural seawater pumped 10 m depth was added in a pressure tank and filtered through a 0.22 μm Sterivex filter (Sterivex™-GV Sterile Vented Filter Unit 0.22 μm, EMD Millipore, Billerica, USA) by positive pressure using filtered (0.22 μm) high purity N_2_ gas. Feces were collected into a 1.5 mL tube using an adopted 5 mL tip and preserved at −80 °C until DNA extraction.

Five liters of the same seawater used in the sea cucumber rearing tank was filtered through a 0.22 μm Sterivex filter by positive pressure using filtered (0.22 μm) N_2_ gas. The Sterivex filter was filled with SET buffer (sucrose 20%, EDTA 50 mM, Tris-HCl 50 mM) and preserved at −80 °C until DNA extraction.

### Microbial DNA extraction

Microbial DNA extraction from sea cucumber feces was performed using the NucleoSpin Soil Kit (MACHEREY-NAGEL, Düren, Germany), according to the manufacture’s protocol. Microbial DNA extraction from seawater was performed using the NucleoSpin Tissue kit (MACHEREY-NAGEL), according to the modified manufacture’s protocol. We used SDS (20%) and proteinase K (20 mg mL^−1^) for pre-lysis instead of buffer T1 and proteinase K. Also, we used 1 mL buffer B3 instead of 200 μL buffer B3.

### 16S rRNA genes pyrosequencing

The hypervariable V1-V2 region of the 16S rRNA gene was amplified by PCR with barcoded 27Fmod and 338R primers^55^. PCR was performed in 50 μL of 1 μL Ex *Taq* PCR buffer composed of 10 mM Tris–HCl (pH 8.3), 50 mM KCl, and 1.5 mM MgCl_2_ in the presence of 250 mM dNTP, 1 U Ex *Taq* polymerase (Takara Bio, Ootsu, Shiga), forward and reverse primers (0.2 μM) and ~20 ng template DNA. Thermal cycling consisted of initial denaturation at 96 °C for 2 min, followed by 25 cycles of denaturation at 96 °C for 30 s, annealing at 55 °C for 45 s and extension at 72 °C for 1 min, and final extension at 72 °C on a 9700 PCR system (Life Technologies Japan, Tokyo, Japan). Negative controls were treated similarly, except that no template DNA was added to the PCR reactions. PCR products of ~370 bp were visualized using electrophoresis on 2% agarose gels, while negative controls failed to produce visible PCR products and were excluded from further analysis. PCR amplicons were purified using AMPure XP magnetic purification beads (Beckman Coulter, Brea, CA, USA), and quantified using the Quant-iT PicoGreen dsDNA Assay Kit (Life Technologies Japan). Equal amounts of each PCR amplicon were mixed and then sequenced using either 454 GS FLX Titanium or 454 GS JUNIOR (Roche, Basel, Switzerland). Based on sample specific barcodes, reads were assigned to each sample followed by the removal of reads lacking both forward and reverse primer sequences. Data was further qualified by removal of reads with average quality values < 25. Filter-passed reads were filed as FASTA for downstream analyses, after trimming off both primer sequences.

### Taxonomic assignment and microbial diversity analyses

Sequences of *Eukarya*, chloroplasts and mitochondria were removed from the dataset using Metaxa software^56^. Sequence analysis was performed using Quantitative Insights Into Microbial Ecology (QIIME) 1.8 software package^57^ as shown below; i) Sequences were clustered into OTUs (similarity 97%) using uclust algorithm^58^. ii) Representative sequences of each OTU were assigned taxonomically using uclust consensus taxonomy assigner with default parameters (minimum percent similarity 90%) and Greengenes reference version 13.8. iii) Sequences were aligned to Greengenes Core reference using PyNAST algorithm^59^ and phylogenic trees were generated using FastTree for tree based analyses. iv) Alpha diversity estimates, observed species, Faith’s phylogenic diversity [PD] and Chao1 were calculated and compared between the larger and the smaller individuals using nonparametric test based on 999 iterations using a rarefaction of 1,835 reads from each sample. v) For beta diversity, even sampling of 1,835 reads per sample was used, and we performed unweighted UniFrac analysis^60^ and visualized in PCoA plots^61^. Beta diversity was compared in a pairwise fashion (larger vs smaller sea cucumbers, larger vs seawater, smaller vs seawater) using unweighted UniFrac distance matrixes, using permutational multivariate analysis of variance (PERMANOVA) with 999 permutations to determine statistical significance. vi) Taxonomic relative abundances of fecal microbial communities between larger and smaller sea cucumbers were compared using Welch’s t test with Storey’s FDR (false discovery rate) for multiple test correction. Significance was assumed to be *P* < 0.05 and *q* < 0.05. Taxonomic affiliations of 16S rRNA gene reads below genus level could not be fully achieved due to the lack of marine microbial reference sequences even in this affiliation setting using QIIME, family table was the lowest hierarchy analyzed in this study (Table S2).

### OTUs relevant to body weight of sea cucumbers

To verify if specific OTUs abundance correlated to sea cucumber body weight, we performed multiple Pearson correlations, and *P* values were adjusted using the Holm method. We searched for highly (*r* > 0.7) and significantly (corrected *P* < 0.05) correlated OTUs against the body weight of the sea cucumbers. OTUs that occurred in only one sample were removed from this analysis. OTUs present in all larger individuals, in all smaller individuals, and all individuals tested were defined as ‘lager individuals core (LIC)’, ‘smaller individuals core (SIC)’, and ‘all individuals core (AIC)’, respectively.

### Metagenomic sequencing

Each representative DNA samples of the largest and the smallest body weight sea cucumbers, from which sufficient volumes were available after 16S typing, were used for metagenome sequencing. Metagenomic sequencing was performed on HiSeq platform by Hokkaido System Science, Co. Ltd., Sapporo, Japan. The DNA quality and quantity were estimated using Nanodrop, Qubit Fluorometer and Agilent 2200 TapeStation System. By using TruSeq Nano DNA LT Sample Prep Kit, genomic DNA was fragment and insert DNA of 350 bp was selected and connected adapter sequences. Sixty nanograms of each DNA sample was amplified with nine cycling. Subsequently, 100 bp paired-end sequencing of two samples was performed on HiSeq platform. Using Chastity, sequences showing low fluorescence purity were removed.

### Pre-processing and data annotation by MG-RAST

Sequencing data set in FASTQ format of samples (the largest and the smallest) was uploaded to MG-RAST server version 3.5. For quality control, firstly, dereplication (removing artificial replicate sequences) was performed using DRISEE (duplicate read inferred sequencing error estimation). Secondly, the removal of low quality sequences was performed by a modified DynamicTrim. After filtering, data gene calling was performed using FragGeneScan algorithm to predict protein or rRNA coding region. Clusters of proteins (similarity 90% or greater) were generated using uclust by BLAT analysis. The sequences assignments were conducted using the MG-RAST server and the following cut-off parameters: expected value less than 1 × 10^−5^, 60% minimum identity and 15 base pair or amino acid minimum alignment. Taxonomic annotation was performed using the National Center for Biotechnology Information (NCBI) GenBank database, and functional annotation was completed using the SEED database^62^.

### Metagenomic analysis

Annotated metagenomes were analyzed using the MG-RAST analysis tools and STAMP software v2.0.9^63^. To test if the abundance of each functional feature of the largest and the smallest sea cucumbers were different, Fisher’s exact test with Newcombe-Wilson confidence interval calculation method and Storey’s FDR for multiple test correction method were used in STAMP software. *P*-values of <0.05 were considered statistically significant. To visualize metagenomic results, profile bar plots and extended error bar plots were generated at Subsystem Level 1 and 3. To plot data, results were filtered by 0.05 of *q*-value and 0.05 of effect size^64^.

To investigate the taxonomic affiliation of the significantly different functional features between the largest and the smallest sea cucumbers, we used the MG-RAST workbench tool implementing KEGG Orthology and GenBank databases to annotate with the functional features.

All sequences generated in this study are deposited in BaMBa^65^ under data package identification number pmeirelles.19.1, and DDBJ/GenBank/EMBL database under BioProject number PRJDB4366.

## Additional Information

**How to cite this article**: Yamazaki, Y. *et al.* Individual *Apostichopus japonicus* fecal microbiome reveals a link with polyhydroxybutyrate producers in host growth gaps. *Sci. Rep.* **6**, 21631; doi: 10.1038/srep21631 (2016).

## Supplementary Material

Supplementary Information
