# Supplementary material for: Individual Apostichopus japonicus fecal microbiome reveals a link with polyhydroxybutyrate producers in host growth gaps
Source: Sci Rep. 2016 Feb 24;6:21631. doi: 10.1038/srep21631 (PMC4764845; doi:10.1038/srep21631)

Individual *Apostichopus japonicus* fecal microbiome reveals a link with polyhydroxybutyrate producers in host growth gaps

Yohei Yamazaki[1#], Pedro Milet Meirelles[2#], Sayaka Mino[1], Wataru Suda[3,4], Kenshiro Oshima[3], Masahira Hattori[3,5], Fabiano L. Thompson[2], Yuichi Sakai[6], Toko Sawabe[7], and Tomoo Sawabe[1*]

[1] Laboratory of Microbiology, Faculty of Fisheries Sciences, Hokkaido University, Hakodate, Japan
[2] Institute of Biology, SAGE-COPPE, Federal University of Rio de Janeiro (UFRJ), Rio de Janeiro, Brazil
[3] Laboratory of Metagenomics, Graduate School of Frontier Sciences, University of Tokyo, Kashiwa, Japan
[4] Department of Microbiology and Immunology, Keio University School of Medicine, Tokyo, Japan
[5] Graduate School of Advanced Science and Engineering, Waseda University, Tokyo, Japan
[6] Hakodate Fisheries Research, Hokkaido Research Organization, Local Independent Administrative Agency, Hakodate, Japan
[7] Department of Food and Nutrition, Hakodate Junior College, Hakodate, Japan

# The aforementioned authors have all contributed equally to this study.

* Corresponding author mailing address: Laboratory of Microbiology, Faculty of Fisheries Sciences, Hokkaido University, 3-1-1 Minato-cho, Hakodate 041-8611, Japan; Telephone: +81-138-40-5569; Facsimile: +81-138-40-5569; e-mail: sawabe@fish.hokudai.ac.jp

SUPPLEMENTAY TABLE S1. Summaries of sequencing information. This table shows the information of 16S rRNA gene and shotgun metagenomic sequencing analysis; sample category, body weight of sea cucumbers, number of total sequenced reads, number of qualified reads, number of qualified reads without eukaryotic reads (16S only), qualified reads length average, Good's coverage (16S only) and number of annotated reads (metagenome only).

Category	Sample ID	Body weight (g)	No. of total reads	No. of reads passed quality check	No. of reads passed quality check (removed eukaryotic reads)	Reads length Average (bp)	16S rDNA Good's coverage (%)	Metagenimic annotated reads
Fecal 16S rRNA gene	Large.01	10	14658	5284	2970	294.74	92.8	-
	Large.02	5.1	13323	5181	3011	294.35	94	-
	Large.03	4	14174	5450	2846	292.82	94.6	-
	Large.04	5.8	14669	5483	4221	298.78	95.3	-
	Large.05	13.6	15153	6026	2800	291.79	92.3	-
	Large.06	3.2	13405	5438	3029	294.23	94.3	-
	Large.07	6.5	16141	5766	2998	293.72	92.9	-
	Large.08	7.2	14379	4838	3709	298.19	94.4	-
	Large.09	4.8	12883	4410	4116	302.33	94.5	-
	Large.10	4.8	13326	5120	3126	295.17	94.4	-
	Small.01	1.3	12520	5464	2005	289.6	92.3	-
	Small.02	1	12270	5053	3826	300.01	94.6	-
	Small.03	1.3	11429	5077	2178	291.54	93.1	-
	Small.04	1.7	11936	5656	1835	289.67	88.1	-
	Small.05	1.2	11661	4959	2403	291.96	94.6	-
	Small.06	1.2	12691	5001	3428	297.49	96	-
	Small.07	0.6	11550	5129	2955	295.04	94	-
	Small.08	0.9	11088	4501	2951	297.12	90.8	-
	Small.09	1.9	12574	5978	1900	289.43	93.1	-
	Small.10	1.1	11600	4865	2709	293.72	94.5	-
Seawater 16S rRNA gene	Water	-	13696	5241	4558	292.24	88.3	-
Fecal metagenomes	Large.05	13.6	22386506	18582516	-	101	-	6049986
	Small.03	1.3	22803396	18156817	-	102	-	4634631

SUPPLEMENTARY TABLE S2. Taxa relative abundances between larger and smaller sea cucumber groups at phylum, class, order and family levels. Comparing taxa relative abundances between larger and smaller individuals by Welch's t test, only taxa with $p < 0.05$ and $q < 0.05$ (Storey's False Discovery Rate) were emphasized bold characters. NA means not applicable. * We could not remove this sequences as Chloroplast by Metaxa software.

Phylum	Larger average	Smaller average	*p*-value	*q*-value
Proteobacteria	**0.574254**	**0.526678**	**0.033636**	**0.001801**
Bacteroidetes	0.340488	0.377979	0.150165	0.003787
Verrucomicrobia	0.012409	0.014875	0.305723	0.004909
Actinobacteria	**0.009633**	**0.015702**	**0.002457**	**0.000301**
Planctomycetes	0.0123	0.00874	0.052154	0.002105
WPS-2	0.006934	0.008117	0.30512	0.004909
Firmicutes	**0.001033**	**0.005035**	**0.037179**	**0.001801**
Fusobacteria	**0.002512**	**0.000143**	**0.031001**	**0.001801**
Chloroflexi	0.001029	0.000667	0.170349	0.003787
WS3	0.000924	0.000675	0.435711	0.005861
Acidobacteria	0.000342	0.000807	0.241274	0.004869
SBR1093	0.00053	0.000612	0.823583	0.009065
Lentisphaerae	0.00066	0.000391	0.474736	0.00605
Cyanobacteria	0.000318	0.000228	0.569334	0.006565
TM6	0.000262	0.000151	0.344626	0.004909
Spirochaetes	**0.000411**	**NA**	**0.002485**	**0.000301**
GN02	9.00E-05	0.000148	0.54818	0.006565
TM7	2.40E-05	0.000134	0.172025	0.003787
Fibrobacteres	0.000135	NA	0.101213	0.003064
Nitrospirae	0.000102	NA	0.081248	0.002811
Tenericutes	NA	4.60E-05	0.343436	0.004909
BRC1	3.30E-05	NA	0.343436	0.004909

SUPPLEMENTARY TABLE S2. Continued.

Class	Larger average	Smaller average	*p*-value	*q*-value
Gammaproteobacteria	0.396819	0.399983	0.863861	0.709386
Flavobacteriia	0.289925	0.334573	0.105446	0.241232
Alphaproteobacteria	**0.15251**	**0.114835**	**0.002643**	**0.034263**
Cytophagia	0.032055	0.031946	0.985793	0.723374
Deltaproteobacteria	**0.023212**	**0.010718**	**0.00128**	**0.034263**
Verrucomicrobiae	0.012148	0.014684	0.287557	0.349374
Acidimicrobiia	0.008656	0.012821	0.008466	0.082314
[Saprospirae]	0.011165	0.006852	0.052657	0.214801
Phycisphaerae	0.005483	0.006919	0.338937	0.349374
Bacteroidia	0.00626	0.003417	0.057595	0.214801
Actinobacteria	0.000865	0.002567	0.019679	0.136074
Clostridia	0.000967	0.002042	0.213715	0.349374
Fusobacteriia	0.002512	0.000143	0.031001	0.172239
Bacilli	6.60E-05	0.002467	0.038086	0.185152
BME43	0.00097	0.000947	0.949545	0.723374
OM190	0.001174	0.000519	0.020993	0.136074
Anaerolineae	0.001029	0.000589	0.083667	0.232423
Epsilonproteobacteria	0.001272	0.000339	0.280662	0.349374
PRR-12	0.000924	0.000675	0.435711	0.413302
Betaproteobacteria	0.000384	0.000694	0.248608	0.349374
[Lentisphaeria]	0.00066	0.000391	0.474736	0.439598
EC214	0.00041	0.000403	0.979941	0.723374
Erysipelotrichi	NA	0.000526	0.121205	0.261879
Sva0725	8.10E-05	0.000358	0.098499	0.241232
Chloroplast	0.000294	0.000134	0.236995	0.349374
Spirochaetes	**0.000411**	**NA**	**0.002485**	**0.034263**
Opitutae	0.000195	0.000191	0.969634	0.723374
SJA-4	0.000262	0.000118	0.216894	0.349374
Thermoleophilia	3.20E-05	0.000313	0.060754	0.214801
Acidobacteria-6	6.30E-05	0.00028	0.221426	0.349374
VHS-B5-50	0.00012	0.000209	0.538714	0.48724
C6	6.80E-05	0.000237	0.179943	0.349374
RB25	0.000132	8.10E-05	0.59026	0.510134
Sphingobacteriia	3.40E-05	0.000148	0.219747	0.349374
[Rhodothermi]	8.00E-05	9.60E-05	0.836451	0.707189
BD1-5	9.00E-05	8.10E-05	0.89722	0.712125
TG3	0.000135	NA	0.101213	0.241232
TA18	2.40E-05	8.40E-05	0.35035	0.349374
TM7-1	2.40E-05	8.10E-05	0.374014	0.363648
Nitrospira	0.000102	NA	0.081248	0.232423
AT-s2-57	6.70E-05	3.40E-05	0.562881	0.497527
4C0d-2	NA	9.40E-05	0.083606	0.232423
OPB41	8.10E-05	NA	0.343436	0.349374
Ellin6529	NA	7.90E-05	0.193872	0.349374
Verruco-5	6.60E-05	NA	0.343436	0.349374
Planctomycetia	2.70E-05	3.40E-05	0.875529	0.709386
BPC102	NA	5.40E-05	0.343436	0.349374
TM7-3	NA	5.30E-05	0.343436	0.349374
Mollicutes	NA	4.60E-05	0.343436	0.349374
SBRH58	NA	3.40E-05	0.343436	0.349374
NPL-UPA2	3.30E-05	NA	0.343436	0.349374
Pla3	NA	2.60E-05	0.343436	0.349374
Oscillatoriophycideae	2.40E-05	NA	0.343436	0.349374

SUPPLEMENTARY TABLE S2. Continued.

Order	Larger average	Smaller average	*p*-value	*q*-value
Flavobacteriales	0.289925	0.334573	0.105446	0.033251
Alteromonadales	0.239184	0.250987	0.538428	0.062472
Rhodobacterales	**0.147582**	**0.111355**	**0.004065**	**0.008427**
Thiotrichales	0.05282	0.049581	0.60935	0.068284
HTCC2188	0.036971	0.030522	0.09492	0.033251
Cytophagales	0.032055	0.031946	0.985793	0.09849
[Marinicellales]	**0.028409**	**0.034792**	**0.040825**	**0.027937**
Vibrionales	0.022384	0.018276	0.661444	0.073134
Verrucomicrobiales	0.012148	0.014684	0.287557	0.047466
Acidimicrobiales	**0.008656**	**0.012821**	**0.008466**	**0.014041**
[Saprospirales]	0.011165	0.006852	0.052657	0.030684
Desulfobacterales	**0.010853**	**0.002262**	**0.00032**	**0.002654**
Oceanospirillales	**0.00913**	**0.003392**	**0.043796**	**0.027937**
Phycisphaerales	0.005483	0.006919	0.338937	0.047466
Bacteroidales	0.00626	0.003417	0.057595	0.030684
Spirobacillales	0.004004	0.002775	0.376571	0.051124
Legionellales	0.002566	0.003504	0.317941	0.047466
Myxococcales	0.002894	0.00256	0.743589	0.07866
NB1-j	**0.002628**	**0.001142**	**0.001965**	**0.006869**
Clostridiales	0.000942	0.002042	0.203179	0.045537
Actinomycetales	**0.000833**	**0.001852**	**0.025095**	**0.025708**
Fusobacteriales	**0.002512**	**0.000143**	**0.031001**	**0.025708**
Chromatiales	0.00103	0.001449	0.434712	0.053524
Rhizobiales	**0.001571**	**0.000752**	**0.040952**	**0.027937**
Lactobacillales	**6.60E-05**	**0.001772**	**0.024059**	**0.025708**
Campylobacterales	0.001272	0.000339	0.280662	0.047466
GN03	9.00E-04	0.000675	0.463427	0.055695
Caldilineales	0.000996	0.000563	0.10472	0.033251
Bdellovibrionales	0.000842	0.000685	0.735661	0.07866
agg27	**0.001047**	**0.000464**	**0.030657**	**0.025708**
Syntrophobacterales	0.000673	0.000783	0.802773	0.083213
Thiohalorhabdales	0.000734	0.000602	0.542419	0.062472
GMD14H09	**0.000938**	**0.000199**	**0.030655**	**0.025708**
Lentisphaerales	0.00066	0.000391	0.474736	0.056239
BD7-3	0.000524	0.000286	0.275597	0.047466
Bifidobacteriales	3.20E-05	0.000716	0.083899	0.033167
Burkholderiales	0.00027	0.000456	0.388403	0.051124
Rickettsiales	0.00031	0.000364	0.831643	0.084589
Kiloniellales	0.000501	0.000157	0.12866	0.036026
HOC36	0.000177	0.00044	0.262759	0.047466
Rhodospirillales	0.000374	0.000223	0.434111	0.053524
Bacillales	NA	0.000566	0.137552	0.036795
Erysipelotrichales	NA	0.000526	0.121205	0.035896
Sva0725	8.10E-05	0.000358	0.098499	0.033251
Spirochaetales	**0.000411**	**NA**	**0.002485**	**0.006869**
Gaiellales	3.20E-05	0.000313	0.060754	0.030684
Pseudomonadales	NA	0.000299	0.062904	0.030684
Caulobacterales	0.000102	0.000165	0.687108	0.074972
Desulfuromonadales	0.000148	0.000109	0.749368	0.07866
Puniceicoccales	6.90E-05	0.000157	0.385716	0.051124
Neisseriales	NA	0.000211	0.108264	0.033251
d113	6.80E-05	0.000141	0.410952	0.053247
BPC015	NA	0.000189	0.130331	0.036026
Sphingobacteriales	3.40E-05	0.000148	0.219747	0.047466
CL500-15	0.000127	5.40E-05	0.436382	0.053524
[Rhodothermales]	8.00E-05	9.60E-05	0.836451	0.084589
Desulfovibrionales	2.40E-05	0.000135	0.438907	0.053524
TG3-2	0.000135	NA	0.101213	0.033251
Gemellales	NA	0.000128	0.083993	0.033167
Opitutales	6.80E-05	3.40E-05	0.550434	0.062527
Nitrospirales	0.000102	NA	0.081248	0.033167
MVS-107	NA	9.60E-05	0.168258	0.040672
MLE1-12	NA	9.40E-05	0.083606	0.033167
CCU21	NA	9.10E-05	0.176569	0.040672
Enterobacteriales	NA	8.40E-05	0.176438	0.040672
PHOS-HD29	NA	8.40E-05	0.176438	0.040672
WCHB1-41	6.60E-05	NA	0.343436	0.047466
iii1-15	6.30E-05	NA	0.172472	0.040672
B110	NA	5.40E-05	0.343436	0.047466
PB19	NA	3.40E-05	0.343436	0.047466
Methylococcales	NA	3.40E-05	0.343436	0.047466
Pasteurellales	NA	3.40E-05	0.343436	0.047466
Pirellulales	NA	3.40E-05	0.343436	0.047466
Desulfarculales	NA	3.40E-05	0.343436	0.047466
S0208	3.40E-05	NA	0.343436	0.047466
UA01	3.30E-05	NA	0.343436	0.047466
Planctomycetales	2.70E-05	NA	0.343436	0.047466
CFB-26	NA	2.60E-05	0.343436	0.047466
A21b	NA	2.60E-05	0.343436	0.047466
OPB54	2.40E-05	NA	0.343436	0.047466
Sediment-1	2.40E-05	NA	0.343436	0.047466
Chroococcales	2.40E-05	NA	0.343436	0.047466
CV90	2.40E-05	NA	0.343436	0.047466

SUPPLEMENTARY TABLE S2. Continued.

Family	Larger average	Smaller average	*p*-value	*q*-value
Flavobacteriaceae	0.250229	0.280026	0.230014	0.182131
OM60	0.210828	0.225557	0.421353	0.213347
Rhodobacteraceae	**0.147582**	**0.111009**	**0.003903**	**0.035572**
Piscirickettsiaceae	0.040435	0.038691	0.714755	0.298823
HTCC2089	0.035504	0.03031	0.168932	0.182131
Flammeovirgaceae	0.031834	0.031638	0.974185	0.37819
[Marinicellaceae]	0.028409	0.034792	0.040825	0.133232
Cryomorphaceae	0.024347	0.028741	0.282993	0.182131
Vibrionaceae	0.017315	0.012131	0.493006	0.234026
Verrucomicrobiaceae	0.012148	0.014684	0.287557	0.182131
C111	0.00726	0.010185	0.01665	0.094844
Desulfobulbaceae	**0.010787**	**0.002157**	**0.000324**	**0.007382**
Alteromonadaceae	0.003809	0.00799	0.201449	0.182131
Pseudoalteromonadaceae	0.005035	0.006145	0.643308	0.281883
Colwelliaceae	0.007007	0.002464	0.11625	0.165104
Oleiphilaceae	0.005111	0.001284	0.030891	0.129302
VC21_Bac22	**0.004885**	**0.00094**	**0.001675**	**0.025444**
Saprospiraceae	0.002302	0.002379	0.951694	0.376905
Moritellaceae	0.000305	0.0041	0.343716	0.182131
Halomonadaceae	0.001983	0.000739	0.034049	0.129302
Fusobacteriaceae	0.00248	0.000143	0.03377	0.129302
Coxiellaceae	0.000483	0.002138	0.050184	0.142932
JTB38	**0.001946**	**0.000314**	**4.70E-05**	**0.002142**
Oceanospirillaceae	0.001637	0.000598	0.262829	0.182131
Micrococcaceae	0.00069	0.001441	0.057879	0.155151
OM27	0.001431	0.00053	0.106266	0.165104
Psychromonadaceae	0.00129	0.000313	0.254449	0.182131
Caldilineaceae	0.000996	0.000563	0.10472	0.165104
Shewanellaceae	0.001249	0.00027	0.117582	0.165104
Phycisphaeraceae	0.000602	0.000904	0.279577	0.182131
Syntrophobacteraceae	0.000673	0.000783	0.802773	0.329574
Streptococcaceae	6.60E-05	0.001359	0.014708	0.094844
Prevotellaceae	NA	0.001398	0.19031	0.182131
KSB4	0.000683	0.000675	0.979285	0.37819
Campylobacteraceae	0.001102	0.000237	0.301831	0.182131
Phyllobacteriaceae	0.000926	0.000377	0.161999	0.182131
JdFBGBact	0.000177	0.000963	0.325498	0.182131
Bacteriovoracaceae	0.000633	0.000427	0.634917	0.280907
Lentisphaeraceae	0.00066	0.000391	0.474736	0.228763
Ruminococcaceae	0.000697	0.000268	0.040931	0.133232
Chitinophagaceae	0.000606	0.000354	0.310907	0.182131
Bifidobacteriaceae	3.20E-05	0.000716	0.083899	0.159483
Peptostreptococcaceae	5.40E-05	0.000669	0.36325	0.188108
Porphyromonadaceae	NA	0.000682	0.235229	0.182131
Chromatiaceae	0.00039	0.000246	0.465198	0.227949
Hyphomicrobiaceae	0.000445	0.000179	0.236738	0.182131
NB1-i	0.000203	0.000409	0.120991	0.165104
koll13	0.000193	0.000397	0.325279	0.182131
Clostridiaceae	0.000126	0.000454	0.111429	0.165104
Oxalobacteraceae	0.000102	0.000456	0.062403	0.156098
SB-1	0.000548	NA	0.007539	0.057259
Erysipelotrichaceae	NA	0.000526	0.121205	0.165104
[Amoebophilaceae]	0.000197	0.000307	0.631993	0.280907
Bdellovibrionaceae	0.000209	0.000257	0.709353	0.298823
Nannocystaceae	3.40E-05	0.000433	0.350907	0.183805
Planococcaceae	NA	0.00042	0.25392	0.182131
Spirochaetaceae	**0.000411**	**NA**	**0.002485**	**0.028311**
Ferrimonadaceae	0.000267	0.000121	0.264981	0.182131
Rickettsiaceae	0.000195	0.000172	0.874925	0.352838
Hyphomonadaceae	NA	0.000347	0.282469	0.182131
Lachnospiraceae	3.20E-05	0.000302	0.123184	0.165104
Legionellaceae	0.000273	4.20E-05	0.04816	0.142932
wb1_P06	0.000236	5.40E-05	0.162629	0.182131
Gaiellaceae	NA	0.00028	0.065083	0.156098
Helicobacteraceae	0.000171	0.000102	0.565695	0.260394
Veillonellaceae	NA	0.000272	0.075565	0.159483
Caulobacteraceae	0.000102	0.000165	0.687108	0.296999
Pseudomonadaceae	NA	0.000265	0.098594	0.165104
Desulfuromonadaceae	0.000148	0.000109	0.749368	0.310446
Kiloniellaceae	0.000229	NA	0.030031	0.129302
Saccharospirillaceae	NA	0.000227	0.137312	0.178782
Puniceicoccaceae	6.90E-05	0.000157	0.385716	0.197497
Neisseriaceae	NA	0.000211	0.108264	0.165104
Marinilabiaceae	6.90E-05	0.000131	0.598183	0.269896
Rhodospirillaceae	0.000137	6.30E-05	0.476899	0.228763
Bacteroidaceae	9.10E-05	0.000108	0.872049	0.352838
Desulfobacteraceae	6.60E-05	0.000105	0.690841	0.296999
Desulfovibrionaceae	2.40E-05	0.000135	0.438907	0.217404
Rhodothermaceae	5.60E-05	9.60E-05	0.597689	0.269896
Staphylococcaceae	NA	0.000146	0.209508	0.182131
Propionibacteriaceae	5.60E-05	8.10E-05	0.710969	0.298823
Burkholderiaceae	0.000135	NA	0.343436	0.182131
Actinomycetaceae	NA	0.000134	0.221429	0.182131
Enterococcaceae	NA	0.000134	0.221429	0.182131
Gemellaceae	NA	0.000128	0.083993	0.159483
Ectothiorhodospiraceae	3.30E-05	8.80E-05	0.437552	0.217404
Lactobacillaceae	NA	0.000112	0.082443	0.159483
Thiotrichaceae	0.000107	NA	0.343436	0.182131
[Weeksellaceae]	NA	0.000107	0.167923	0.182131
Opitutaceae	6.80E-05	3.40E-05	0.550434	0.258593
Nitrospiraceae	0.000102	NA	0.081248	0.159483
HTCC2188	5.10E-05	4.60E-05	0.934792	0.373675
Bradyrhizobiaceae	3.20E-05	6.30E-05	0.565131	0.260394
Enterobacteriaceae	NA	8.40E-05	0.176438	0.182131
Carnobacteriaceae	NA	6.80E-05	0.343436	0.182131
NS11-12	3.40E-05	3.40E-05	0.996429	0.381578
Mycobacteriaceae	NA	6.00E-05	0.171764	0.182131
S24-7	NA	5.20E-05	0.343436	0.182131
Pseudonocardiaceae	2.40E-05	2.60E-05	0.959415	0.376905
[Paraprevotellaceae]	NA	5.00E-05	0.343436	0.182131
[Mogibacteriaceae]	NA	5.00E-05	0.343436	0.182131
Corynebacteriaceae	3.60E-05	NA	0.343436	0.182131
Francisellaceae	NA	3.40E-05	0.343436	0.182131
Pasteurellaceae	NA	3.40E-05	0.343436	0.182131
Moraxellaceae	NA	3.40E-05	0.343436	0.182131
Pirellulaceae	NA	3.40E-05	0.343436	0.182131
Desulfarculaceae	NA	3.40E-05	0.343436	0.182131
Comamonadaceae	3.30E-05	NA	0.343436	0.182131
MND4	3.30E-05	NA	0.343436	0.182131
Leptotrichiaceae	3.20E-05	NA	0.343436	0.182131
Microthrixaceae	2.70E-05	NA	0.343436	0.182131
Planctomycetaceae	2.70E-05	NA	0.343436	0.182131
Nocardioidaceae	NA	2.60E-05	0.343436	0.182131
EB1003	NA	2.60E-05	0.343436	0.182131
[Balneolaceae]	2.40E-05	NA	0.343436	0.182131
PRR-10	2.40E-05	NA	0.343436	0.182131
Cyclobacteriaceae	2.40E-05	NA	0.343436	0.182131
Xenococcaceae	2.40E-05	NA	0.343436	0.182131
Methylobacteriaceae	2.40E-05	NA	0.343436	0.182131

SUPPLEMENTARY TABLE S3. OUTs showing strong correlations with *Apostichopus japonicus* body weight. Correlation coefficient was calculated by Pearson. Significance was justified by holm adjusted *p*-value. Numbers in parentheses mean the numbers of individuals sharing the OTUs, and L, S and W mean Larger individuals, smaller individuals and seawater sample, respectively.

OTU	Correlation coefficient	Adjusted *p*-value	Phylum	Class	Order	Family	Genus	Affiliation
585	0.816536	0.001271	*Proteobacteria*	*Gammaproteobacteria*	*Legionellales*	*Legionellaceae*		Larger only (5)
1884	0.800120	0.002555	*Proteobacteria*	*Gammaproteobacteria*	*Alteromonadales*	*Alteromonadaceae*	*BD2-13*	Larger only (3)
2268	0.796084	0.002984	*Bacteroidetes*	*Flavobacteriia*	*Flavobacteriales*	*Cryomorphaceae*		Larger only (8)
1150	0.791885	0.003494	*Proteobacteria*	*Gammaproteobacteria*	*Alteromonadales*	*OM60*		All samples
268	0.786718	0.004231	*Proteobacteria*	*Deltaproteobacteria*	*GMD14H09*			Larger only (2)
451	0.758249	0.011562	*Proteobacteria*	*Gammaproteobacteria*	*Vibrionales*	*Vibrionaceae*		N (L:3, W)
2068	0.758050	0.011531	*Proteobacteria*	*Gammaproteobacteria*	*Alteromonadales*			LIC
289	0.749621	0.015030	*Proteobacteria*	*Gammaproteobacteria*	*Vibrionales*	*Vibrionaceae*		N (L:5, S:1)
845	0.727699	0.029007	*Bacteroidetes*	*Cytophagia*	*Cytophagales*	*Flammeovirgaceae*	*Roseivirga*	N (L:8, S:2)
1298	0.717973	0.037876	Unassigned					Larger only (4)
1956	0.715266	0.040431	*Proteobacteria*	*Gammaproteobacteria*	*Alteromonadales*	*Colwelliaceae*		Larger only (3)
494	0.714639	0.040735	*Proteobacteria*	*Gammaproteobacteria*	*Vibrionales*	*Vibrionaceae*	*Aliivibrio* (*fischeri*)	N (L:8, S:1, W)
1373	0.711881	0.043491	*Bacteroidetes*	*Flavobacteriia*	*Flavobacteriales*	*Flavobacteriaceae*		N (L:9, S:1, W)
1353	0.711281	0.043767	Unassigned					LIC
1762	0.710193	0.044622	*Proteobacteria*	*Gammaproteobacteria*	*Vibrionales*	*Vibrionaceae*	*Photobacterium* (*rosenbergii*)	N (L:6, S:2)

SUPPLEMENTARY FIG. S1. Core fecal microbitota of cultured junvenile *Apostichopus japonicus*. (**a**) Venn diagram of core microbiota, and (**b**) the order level bacterial structure.

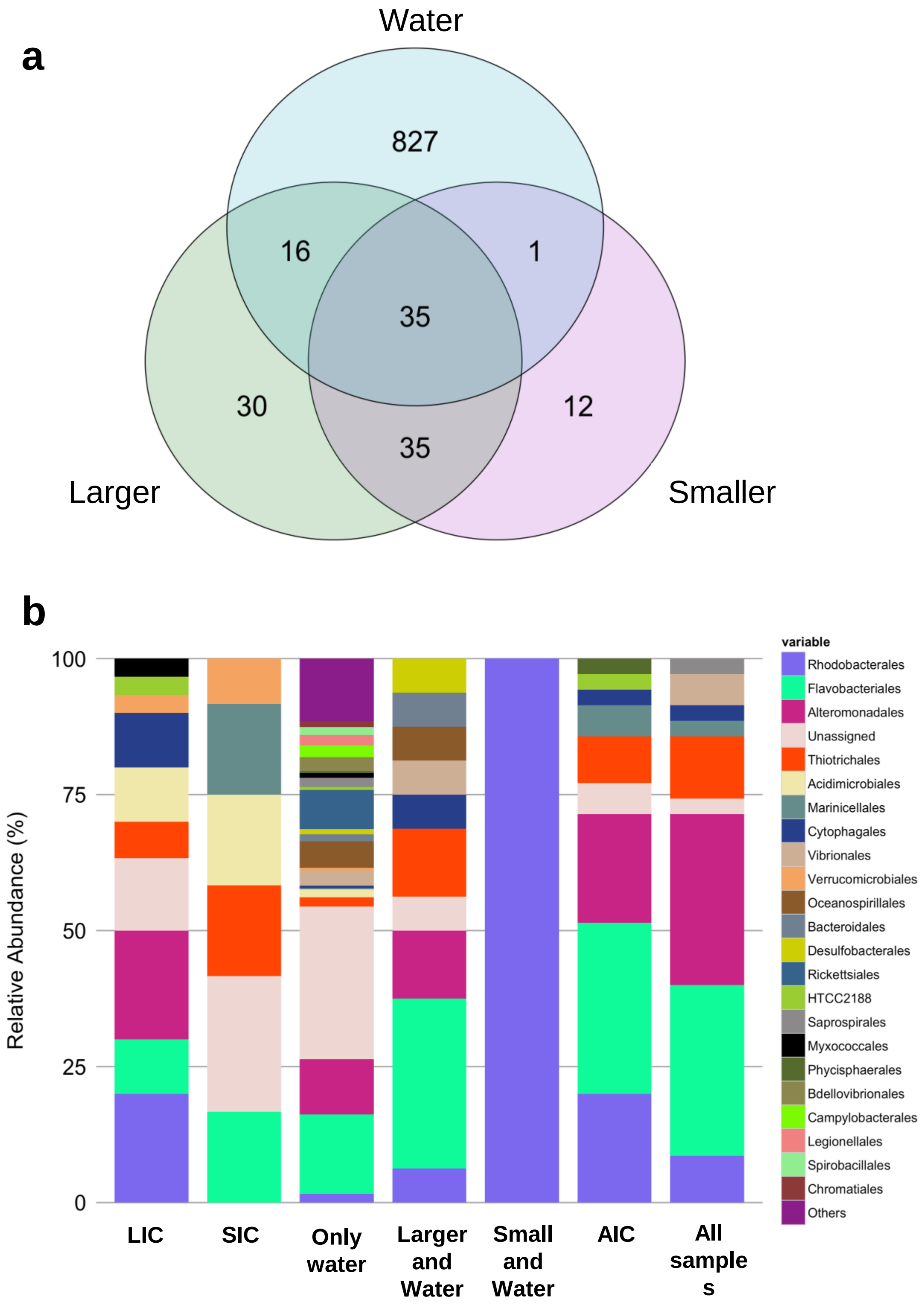

SUPPLEMENTARY FIG. S2. Comparative metagenomic analyses at subsystem level 1 and 3. (**a**) Bar plot shows proportions of sequences for each functional feature at subsystem level 1. The largest indicated by green and the smallest indicated by purple color. Significantly different proportion of a feature between groups is indicated by an asterisk, respectively. (**b**) Only more abundant features in the smallest sea cucumbers are displayed. Green bars indicate the largest sea cucumber and purple indicate the smallest sea cucumber. Proportion (left side) means a possible abundance of microbes possessing each functional feature, and difference between proportions (= effect sizes) for each feature is indicated by a green dot. For this analysis, features were filtered by q value < 0.05 and effect size < 0.05. These functions were associated with ribosome, RNA polymerase and photosystems.

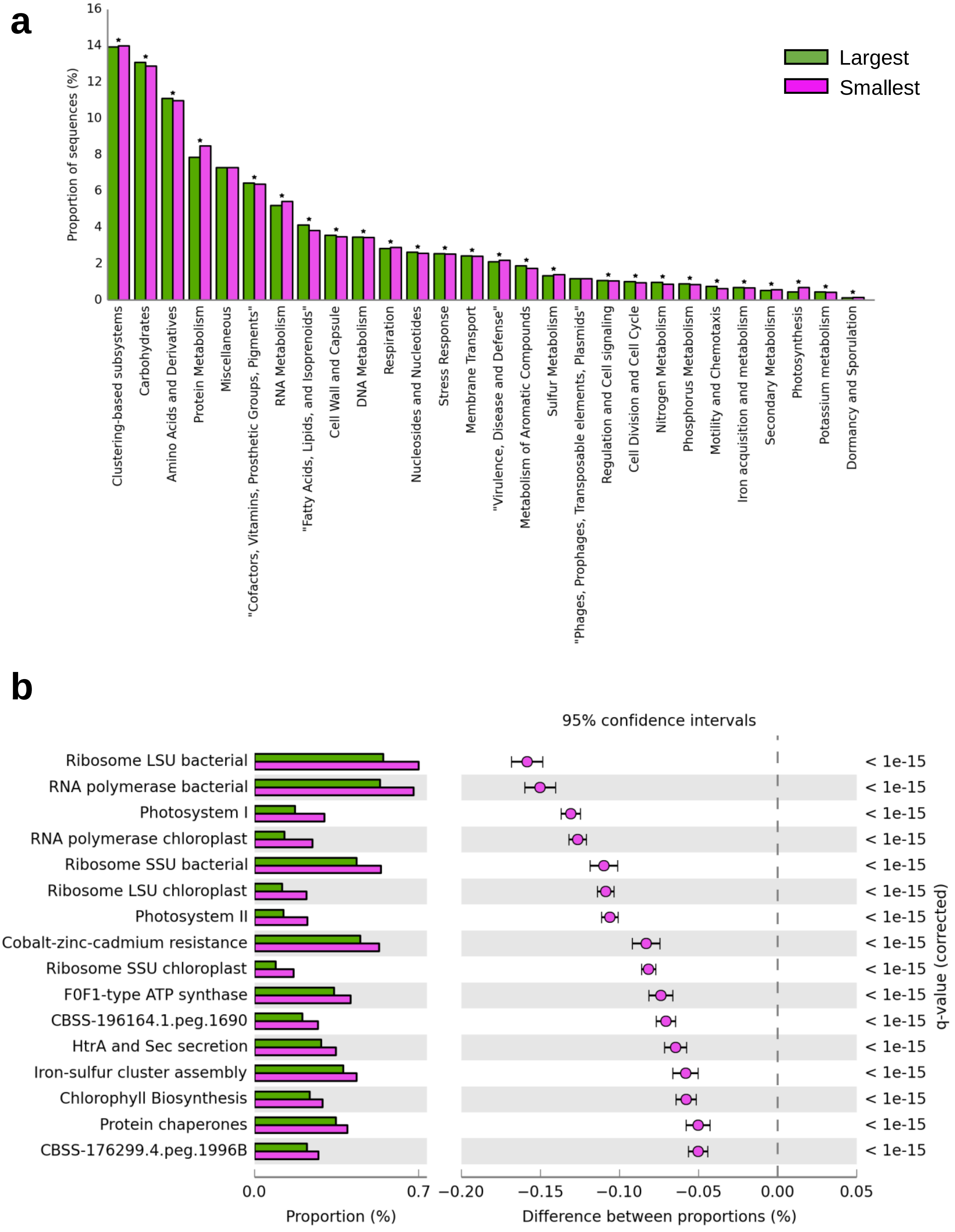

SUPPLEMENTARY FIG. S3. KEGG mapping of megtagenomic reads. Calculating the difference of read proportions between the largest and the smallest individuals for each gene, abundant genes in the largest individual was described by yellow color, conversely abundant genes in the smallest individual was described by blue color. Greater different genes between individuals were colored brighter. (**a**) PHB metabolism–annotated reads are mapped on KEGG pathway. The reads annotated as PHB metabolism in SEED-Subsystems were re-annotated by KEGG ORTHOLOG and mapped on butanoate metabolism pathway. The reads were mapped on genes required to synthesize PHB. Acetyl-CoA C-acetyltransferase (EC:2.3.1.9), 3-hydroxybutyryl-CoA epimerase (EC:5.1.2.3) and polyhydroxyalkanoate synthase (EC:2.3.1.-) were more abundant in the largest individual, conversely 3-hydroxybutyryl-CoA dehydrogenase (EC:1.1.1.157) was more abundant in the smallest individual. (**b**) n-Phenylalkanoic acid degradation, (**c**) fatty acid metabolism cluster, (**d**) acetyl-CoA fermentation to butyrate, (**e**) Fatty acid degradation regulons, (**f**) serine-glyoxylate cycle, (**g**) butyrate metabolism cluster.

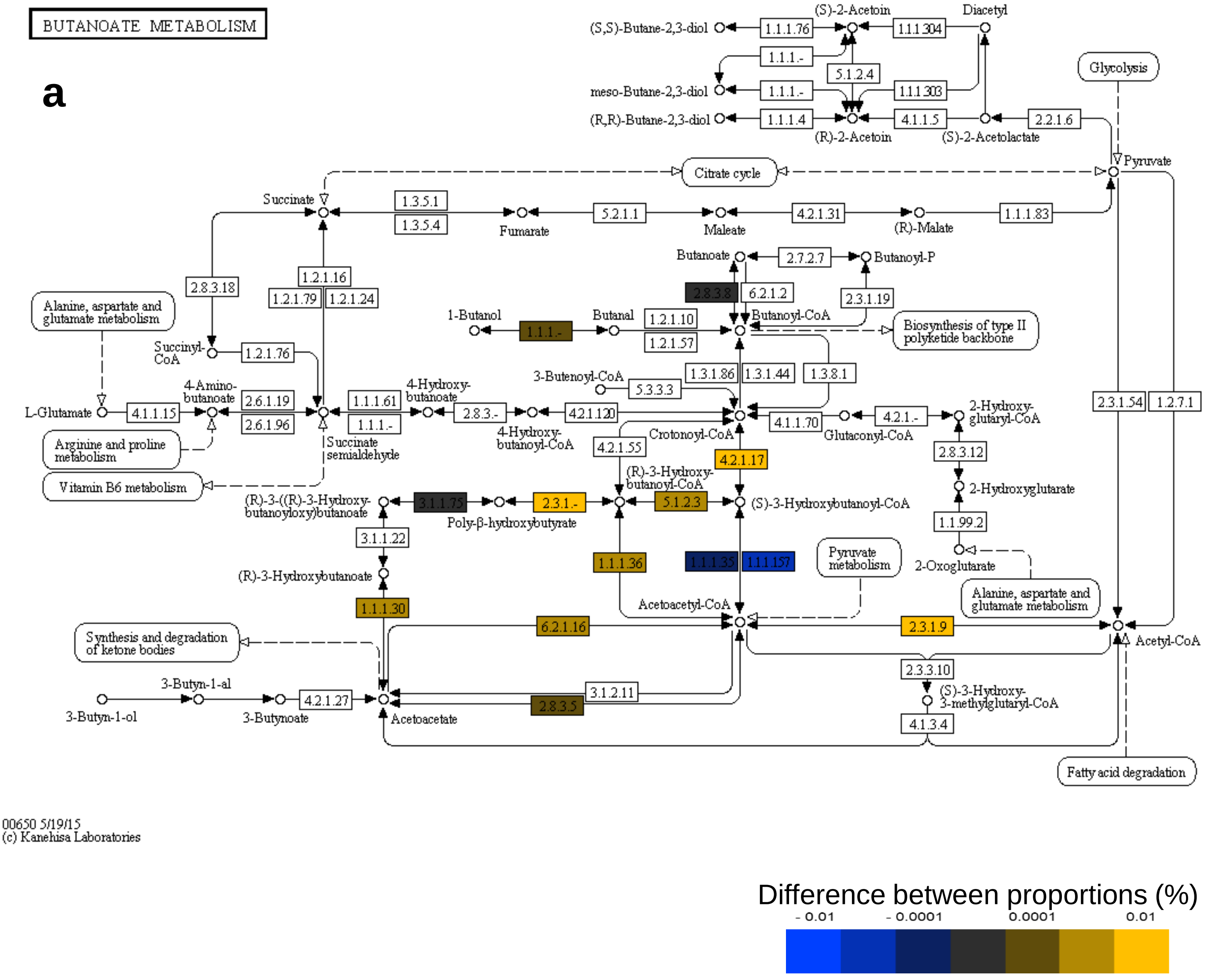

b

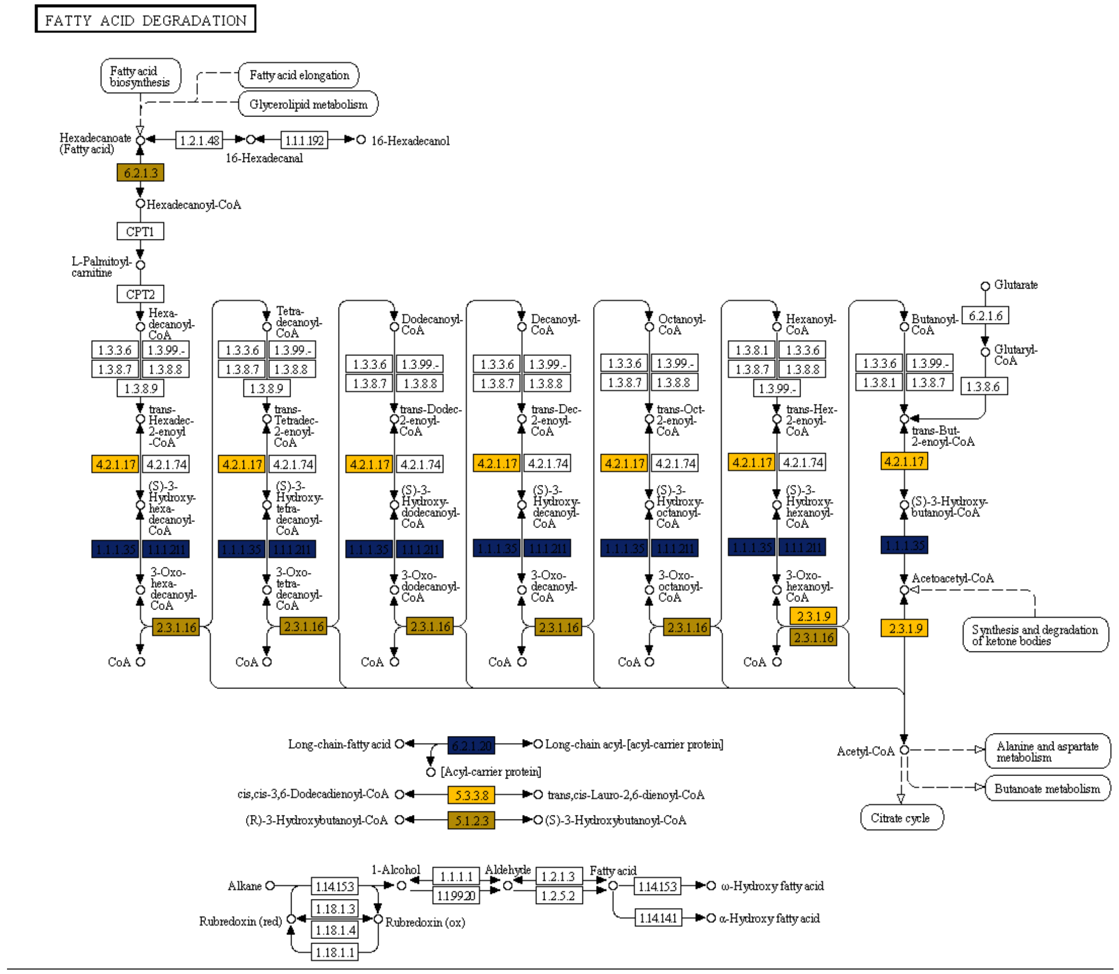

FATTY ACID DEGRADATION
Fatty acid biosynthesis
Fatty acid elongation
Glycerolipid metabolism
Hexadecanoate (Fatty acid)
1.2.1.48
1.1.1.192
16-Hexadecanol
16-Hexadecanal
6.2.1.3
Hexadecanoyl-CoA
CPT1
L-Palmitoyl-carnitine
CPT2
Hexa-decanoyl-CoA
Tetra-decanoyl-CoA
Dodecanoyl-CoA
Decanoyl-CoA
Octanoyl-CoA
Hexanoyl-CoA
Butanoyl-CoA
Glutarate
6.2.1.6
Glutaryl-CoA
1.3.8.6
1.3.3.6
1.3.99.-
1.3.8.7
1.3.8.8
1.3.8.9
1.3.8.1
trans-Hexadec-2-enoyl-CoA
trans-Tetradec-2-enoyl-CoA
trans-Dodec-2-enoyl-CoA
trans-Dec-2-enoyl-CoA
trans-Oct-2-enoyl-CoA
trans-Hex-2-enoyl-CoA
trans-But-2-enoyl-CoA
4.2.1.17
4.2.1.74
(S)-3-Hydroxy-hexa-decanoyl-CoA
(S)-3-Hydroxy-tetra-decanoyl-CoA
(S)-3-Hydroxy-dodecanoyl-CoA
(S)-3-Hydroxy-decanoyl-CoA
(S)-3-Hydroxy-octanoyl-CoA
(S)-3-Hydroxy-hexanoyl-CoA
(S)-3-Hydroxy-butanoyl-CoA
1.1.1.35
1.1.1.211
3-Oxo-hexa-decanoyl-CoA
3-Oxo-tetra-decanoyl-CoA
3-Oxo-dodecanoyl-CoA
3-Oxo-decanoyl-CoA
3-Oxo-octanoyl-CoA
3-Oxo-hexanoyl-CoA
Acetoacetyl-CoA
2.3.1.16
2.3.1.9
CoA
Synthesis and degradation of ketone bodies
Acetyl-CoA
Alanine and aspartate metabolism
Butanoate metabolism
Citrate cycle
Long-chain-fatty acid
6.2.1.20
Long-chain acyl-[acyl-carrier protein]
[Acyl-carrier protein]
cis,cis-3,6-Dodecadienoyl-CoA
5.3.3.8
trans,cis-Lauro-2,6-dienoyl-CoA
(R)-3-Hydroxybutanoyl-CoA
5.1.2.3
(S)-3-Hydroxybutanoyl-CoA
Alkane
1.14.15.3
1-Alcohol
1.1.1.1
1.1.99.20
Aldehyde
1.2.1.3
1.2.5.2
Fatty acid
ω-Hydroxy fatty acid
1.14.14.1
α-Hydroxy fatty acid
1.18.1.3
1.18.1.4
1.18.1.1
Rubredoxin (red)
Rubredoxin (ox)

c

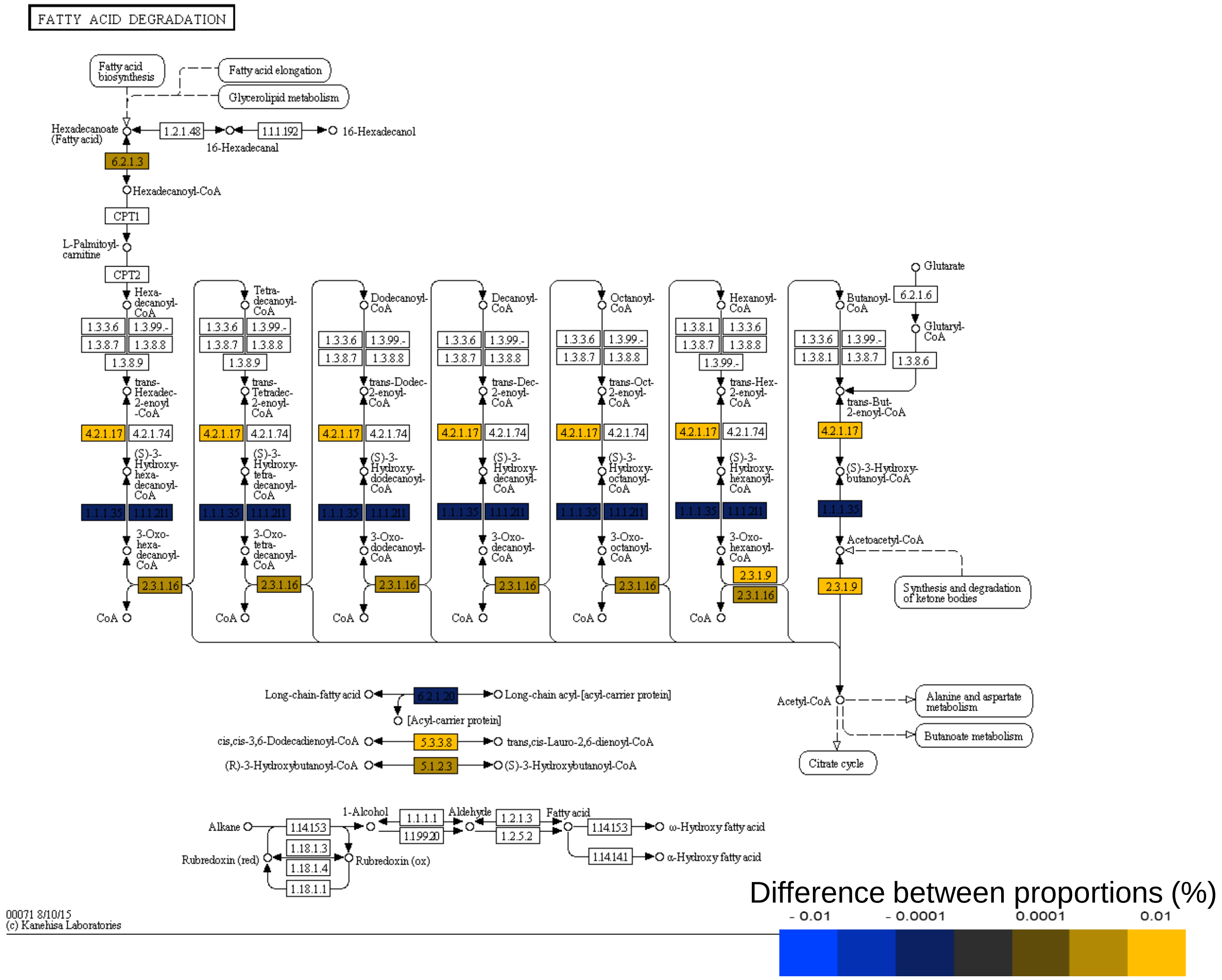

FATTY ACID DEGRADATION
Fatty acid biosynthesis
Fatty acid elongation
Glycerolipid metabolism
Hexadecanoate (Fatty acid)
1.2.1.48
1.1.1.192
16-Hexadecanol
16-Hexadecanal
6.2.1.3
Hexadecanoyl-CoA
CPT1
L-Palmitoyl-carnitine
CPT2
Hexa-decanoyl-CoA
Tetra-decanoyl-CoA
Dodecanoyl-CoA
Decanoyl-CoA
Octanoyl-CoA
Hexanoyl-CoA
Butanoyl-CoA
Glutarate
6.2.1.6
Glutaryl-CoA
1.3.8.6
1.3.3.6
1.3.99.-
1.3.8.7
1.3.8.8
1.3.8.9
1.3.8.1
trans-Hexadec-2-enoyl-CoA
trans-Tetradec-2-enoyl-CoA
trans-Dodec-2-enoyl-CoA
trans-Dec-2-enoyl-CoA
trans-Oct-2-enoyl-CoA
trans-Hex-2-enoyl-CoA
trans-But-2-enoyl-CoA
4.2.1.17
4.2.1.74
(S)-3-Hydroxy-hexa-decanoyl-CoA
(S)-3-Hydroxy-tetra-decanoyl-CoA
(S)-3-Hydroxy-dodecanoyl-CoA
(S)-3-Hydroxy-decanoyl-CoA
(S)-3-Hydroxy-octanoyl-CoA
(S)-3-Hydroxy-hexanoyl-CoA
(S)-3-Hydroxy-butanoyl-CoA
1.1.1.35
1.1.1.211
3-Oxo-hexa-decanoyl-CoA
3-Oxo-tetra-decanoyl-CoA
3-Oxo-dodecanoyl-CoA
3-Oxo-decanoyl-CoA
3-Oxo-octanoyl-CoA
3-Oxo-hexanoyl-CoA
Acetoacetyl-CoA
2.3.1.16
2.3.1.9
CoA
Synthesis and degradation of ketone bodies
Acetyl-CoA
Alanine and aspartate metabolism
Butanoate metabolism
Citrate cycle
Long-chain-fatty acid
6.2.1.20
Long-chain acyl-[acyl-carrier protein]
[Acyl-carrier protein]
cis,cis-3,6-Dodecadienoyl-CoA
5.3.3.8
trans,cis-Lauro-2,6-dienoyl-CoA
(R)-3-Hydroxybutanoyl-CoA
5.1.2.3
(S)-3-Hydroxybutanoyl-CoA
Alkane
1.14.15.3
1-Alcohol
1.1.1.1
1.1.99.20
Aldehyde
1.2.1.3
1.2.5.2
Fatty acid
ω-Hydroxy fatty acid
1.14.14.1
α-Hydroxy fatty acid
1.18.1.3
1.18.1.4
1.18.1.1
Rubredoxin (red)
Rubredoxin (ox)
00071 8/10/15
(c) Kanehisa Laboratories
Difference between proportions (%)
- 0.01
- 0.0001
0.0001
0.01

d

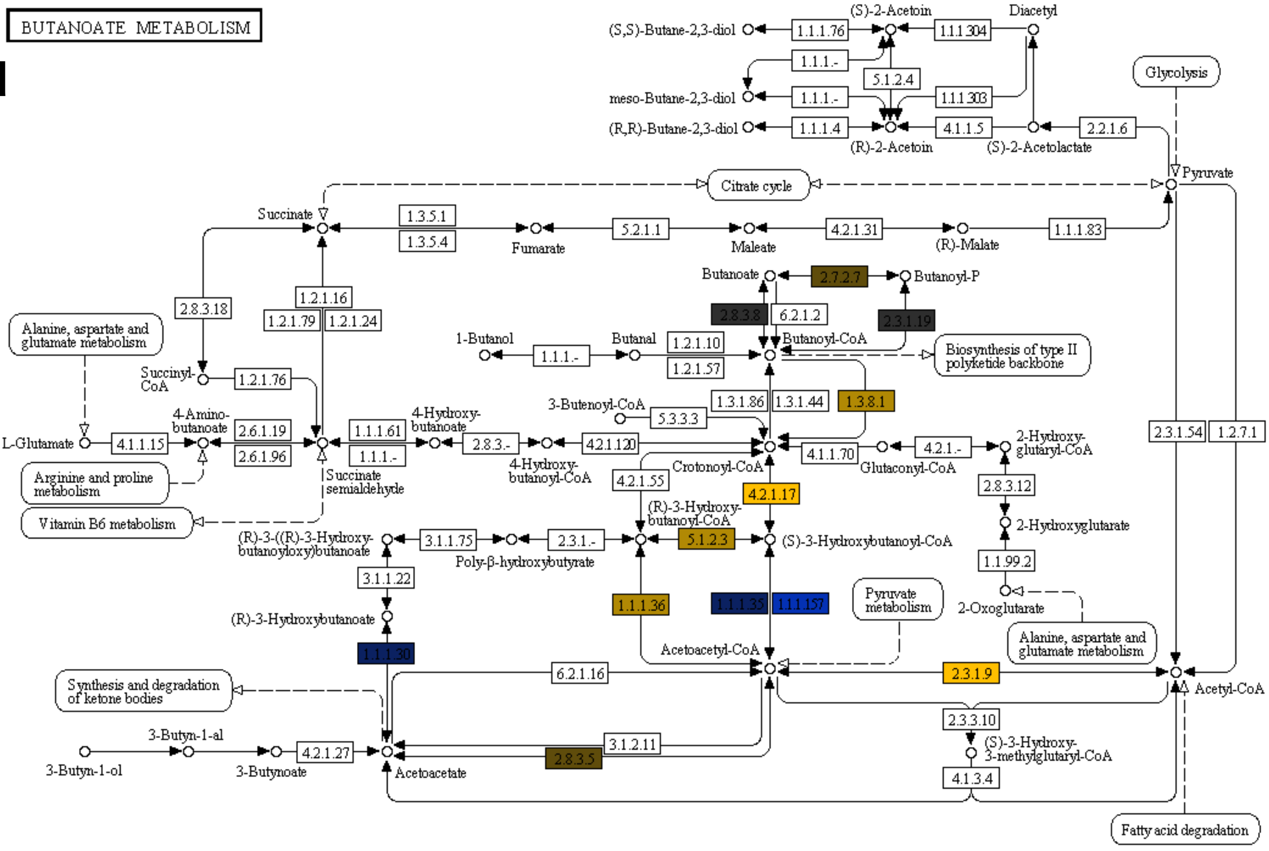
BUTANOATE METABOLISM
(S)-2-Acetoin
Diacetyl
(S,S)-Butane-2,3-diol
1.1.1.76
1.1.1.304
1.1.1.-
5.1.2.4
meso-Butane-2,3-diol
1.1.1.303
(R,R)-Butane-2,3-diol
1.1.1.4
4.1.1.5
2.2.1.6
(R)-2-Acetoin
(S)-2-Acetolactate
Glycolysis
Pyruvate
Citrate cycle
Succinate
1.3.5.1
1.3.5.4
Fumarate
5.2.1.1
Maleate
4.2.1.31
(R)-Malate
1.1.1.83
Butanoate
2.7.2.7
Butanoyl-P
2.8.3.18
1.2.1.16
1.2.1.79
1.2.1.24
2.8.3.8
6.2.1.2
2.3.1.19
Alanine, aspartate and glutamate metabolism
1-Butanol
Butanal
1.2.1.10
1.2.1.57
Butanoyl-CoA
Biosynthesis of type II polyketide backbone
Succinyl-CoA
1.2.1.76
1.3.1.86
1.3.1.44
1.3.8.1
3-Butenoyl-CoA
5.3.3.3
4-Amino-butanoate
4-Hydroxy-butanoate
L-Glutamate
4.1.1.15
2.6.1.19
2.6.1.96
1.1.1.61
2.8.3.-
4.2.1.120
4-Hydroxy-butanoyl-CoA
Succinate semialdehyde
Crotonoyl-CoA
4.1.1.70
4.2.1.-
2-Hydroxy-glutaryl-CoA
Glutaconyl-CoA
Arginine and proline metabolism
4.2.1.55
4.2.1.17
2.8.3.12
2.3.1.54
1.2.7.1
Vitamin B6 metabolism
(R)-3-Hydroxy-butanoyl-CoA
2-Hydroxyglutarate
(R)-3-((R)-3-Hydroxy-butanoyloxy)butanoate
3.1.1.75
2.3.1.-
Poly-β-hydroxybutyrate
5.1.2.3
(S)-3-Hydroxybutanoyl-CoA
1.1.99.2
3.1.1.22
1.1.1.36
1.1.1.35
1.1.1.157
Pyruvate metabolism
2-Oxoglutarate
(R)-3-Hydroxybutanoate
Acetoacetyl-CoA
1.1.1.30
Synthesis and degradation of ketone bodies
6.2.1.16
2.3.1.9
Acetyl-CoA
2.3.3.10
(S)-3-Hydroxy-3-methylglutaryl-CoA
3-Butyn-1-al
3.1.2.11
4.2.1.27
2.8.3.5
3-Butyn-1-ol
3-Butynoate
Acetoacetate
4.1.3.4
Fatty acid degradation

e

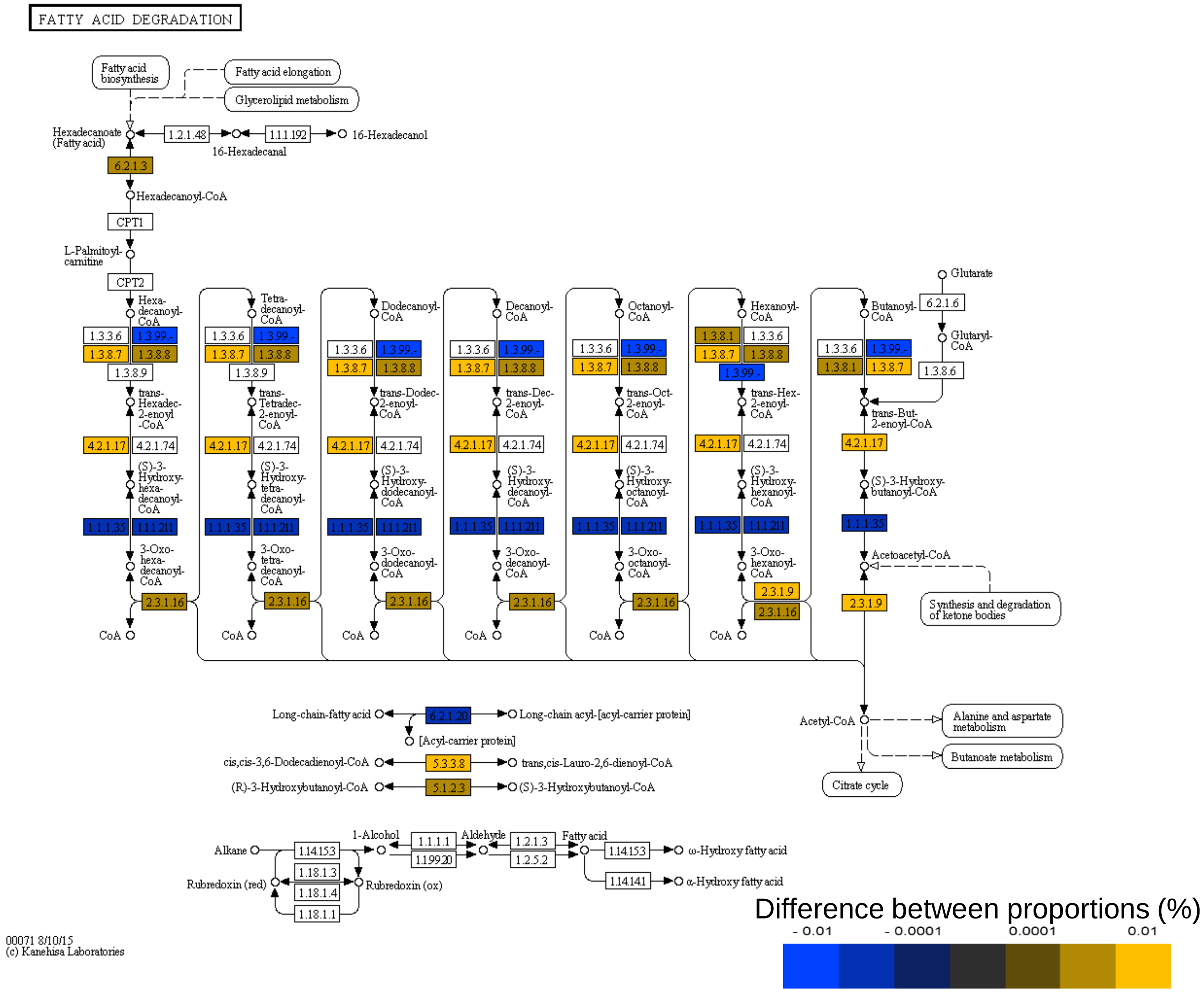
FATTY ACID DEGRADATION
Fatty acid biosynthesis
Fatty acid elongation
Glycerolipid metabolism
Hexadecanoate (Fatty acid)
1.2.1.48
1.1.1.192
16-Hexadecanol
16-Hexadecanal
6.2.1.3
Hexadecanoyl-CoA
CPT1
L-Palmitoyl-carnitine
CPT2
Hexa-decanoyl-CoA
Tetra-decanoyl-CoA
Dodecanoyl-CoA
Decanoyl-CoA
Octanoyl-CoA
Hexanoyl-CoA
Butanoyl-CoA
Glutarate
6.2.1.6
Glutaryl-CoA
1.3.3.6
1.3.99.-
1.3.8.7
1.3.8.8
1.3.8.9
1.3.8.1
1.3.8.6
trans-Hexadec-2-enoyl-CoA
trans-Tetradec-2-enoyl-CoA
trans-Dodec-2-enoyl-CoA
trans-Dec-2-enoyl-CoA
trans-Oct-2-enoyl-CoA
trans-Hex-2-enoyl-CoA
trans-But-2-enoyl-CoA
4.2.1.17
4.2.1.74
(S)-3-Hydroxy-hexa-decanoyl-CoA
(S)-3-Hydroxy-tetra-decanoyl-CoA
(S)-3-Hydroxy-dodecanoyl-CoA
(S)-3-Hydroxy-decanoyl-CoA
(S)-3-Hydroxy-octanoyl-CoA
(S)-3-Hydroxy-hexanoyl-CoA
(S)-3-Hydroxy-butanoyl-CoA
1.1.1.35
1.1.1.211
3-Oxo-hexa-decanoyl-CoA
3-Oxo-tetra-decanoyl-CoA
3-Oxo-dodecanoyl-CoA
3-Oxo-decanoyl-CoA
3-Oxo-octanoyl-CoA
3-Oxo-hexanoyl-CoA
Acetoacetyl-CoA
2.3.1.16
2.3.1.9
Synthesis and degradation of ketone bodies
CoA
Long-chain-fatty acid
6.2.1.20
Long-chain acyl-[acyl-carrier protein]
[Acyl-carrier protein]
Acetyl-CoA
Alanine and aspartate metabolism
Butanoate metabolism
cis,cis-3,6-Dodecadienoyl-CoA
5.3.3.8
trans,cis-Lauro-2,6-dienoyl-CoA
(R)-3-Hydroxybutanoyl-CoA
5.1.2.3
(S)-3-Hydroxybutanoyl-CoA
Citrate cycle
1-Alcohol
Aldehyde
Fatty acid
Alkane
1.14.15.3
1.1.1.1
1.1.99.20
1.2.1.3
1.2.5.2
1.14.15.3
ω-Hydroxy fatty acid
1.18.1.3
1.18.1.4
1.18.1.1
1.14.14.1
α-Hydroxy fatty acid
Rubredoxin (red)
Rubredoxin (ox)
00071 8/10/15
(c) Kanehisa Laboratories
Difference between proportions (%)
- 0.01
- 0.0001
0.0001
0.01

f

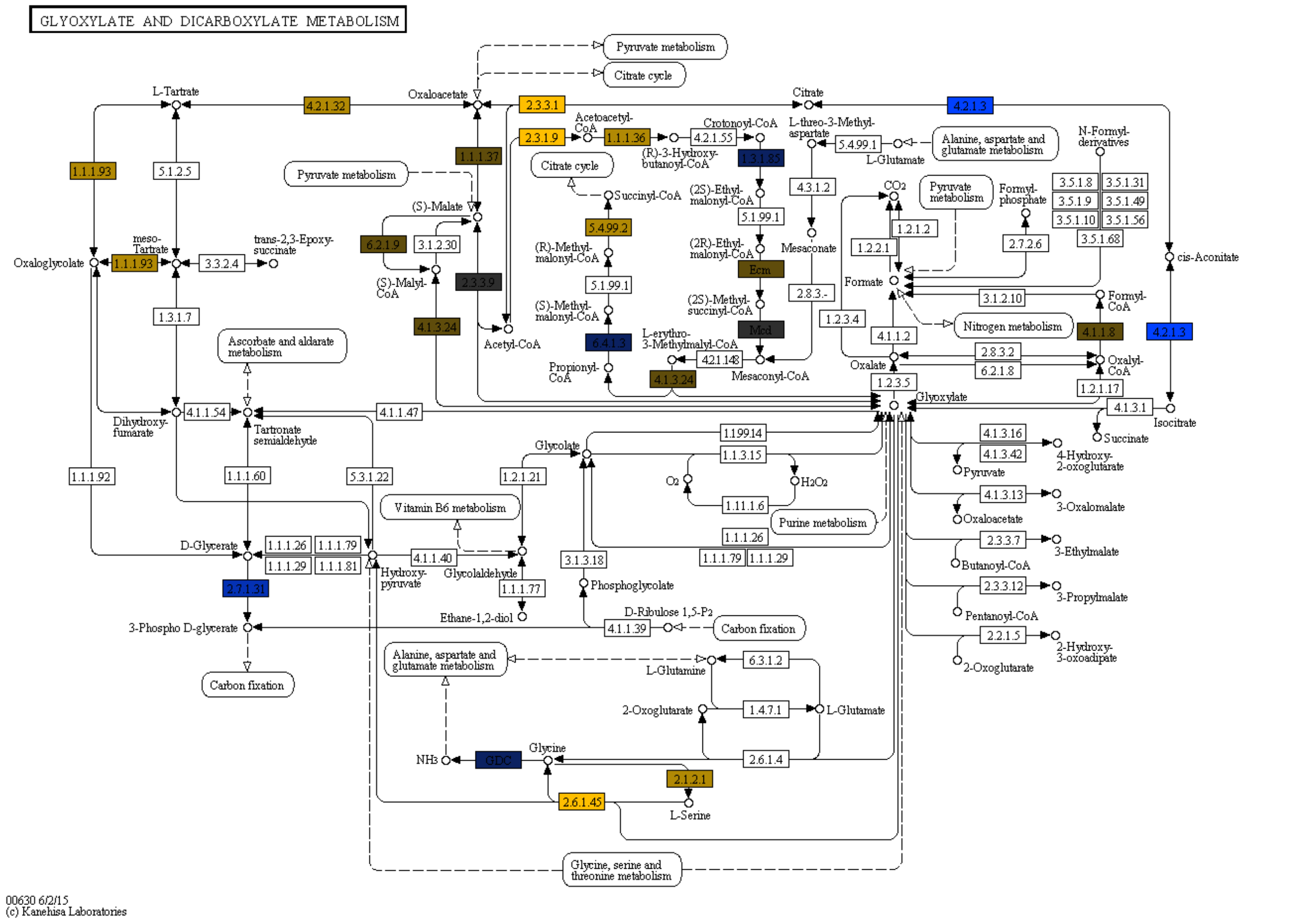
GLYOXYLATE AND DICARBOXYLATE METABOLISM
Pyruvate metabolism
Citrate cycle
L-Tartrate
4.2.1.32
Oxaloacetate
2.3.3.1
Citrate
4.2.1.3
1.1.1.93
5.1.2.5
Pyruvate metabolism
1.1.1.37
2.3.1.9
Acetoacetyl-CoA
1.1.1.36
(R)-3-Hydroxy-butanoyl-CoA
4.2.1.55
Crotonoyl-CoA
1.3.1.85
L-threo-3-Methyl-aspartate
5.4.99.1
L-Glutamate
Alanine, aspartate and glutamate metabolism
N-Formyl-derivatives
Citrate cycle
(S)-Malate
Succinyl-CoA
(2S)-Ethyl-malonyl-CoA
4.3.1.2
CO2
Pyruvate metabolism
Formyl-phosphate
3.5.1.8
3.5.1.31
3.5.1.9
3.5.1.49
3.5.1.10
3.5.1.56
3.5.1.68
meso-Tartrate
trans-2,3-Epoxy-succinate
6.2.1.9
3.1.2.30
5.4.99.2
5.1.99.1
1.2.1.2
2.7.2.6
Oxaloglycolate
1.1.1.93
3.3.2.4
(R)-Methyl-malonyl-CoA
(2R)-Ethyl-malonyl-CoA
Mesaconate
1.2.2.1
cis-Aconitate
(S)-Malyl-CoA
2.3.3.9
Ecm
Formate
5.1.99.1
2.8.3.-
3.1.2.10
Formyl-CoA
1.3.1.7
4.1.3.24
Acetyl-CoA
(S)-Methyl-malonyl-CoA
(2S)-Methyl-succinyl-CoA
1.2.3.4
Nitrogen metabolism
Ascorbate and aldarate metabolism
6.4.1.3
L-erythro-3-Methylmalyl-CoA
Mcd
4.1.1.2
4.1.1.8
4.2.1.3
4.2.1.148
Propionyl-CoA
Oxalate
2.8.3.2
Oxalyl-CoA
4.1.3.24
Mesaconyl-CoA
6.2.1.8
1.2.3.5
1.2.1.17
Dihydroxy-fumarate
4.1.1.54
Tartronate semialdehyde
4.1.1.47
Glyoxylate
4.1.3.1
Isocitrate
1.1.99.14
4.1.3.16
Succinate
Glycolate
1.1.3.15
4.1.3.42
4-Hydroxy-2-oxoglutarate
Pyruvate
1.1.1.92
1.1.1.60
5.3.1.22
1.2.1.21
O2
H2O2
1.1.1.16
4.1.3.13
3-Oxalomalate
Oxaloacetate
Vitamin B6 metabolism
Purine metabolism
1.1.1.26
2.3.3.7
3-Ethylmalate
D-Glycerate
1.1.1.26
1.1.1.79
1.1.1.29
1.1.1.81
4.1.1.40
Hydroxy-pyruvate
Glycolaldehyde
3.1.3.18
1.1.1.79
1.1.1.29
Butanoyl-CoA
2.7.1.31
1.1.1.77
Phosphoglycolate
2.3.3.12
3-Propylmalate
Ethane-1,2-diol
D-Ribulose 1,5-P2
Pentanoyl-CoA
3-Phospho D-glycerate
4.1.1.39
Carbon fixation
2.2.1.5
2-Hydroxy-3-oxoadipate
2-Oxoglutarate
Alanine, aspartate and glutamate metabolism
6.3.1.2
L-Glutamine
Carbon fixation
2-Oxoglutarate
1.4.7.1
L-Glutamate
Glycine
NH3
GDC
2.6.1.4
2.1.2.1
2.6.1.45
L-Serine
Glycine, serine and threonine metabolism
00630 6/2/15
(c) Kanehisa Laboratories

g

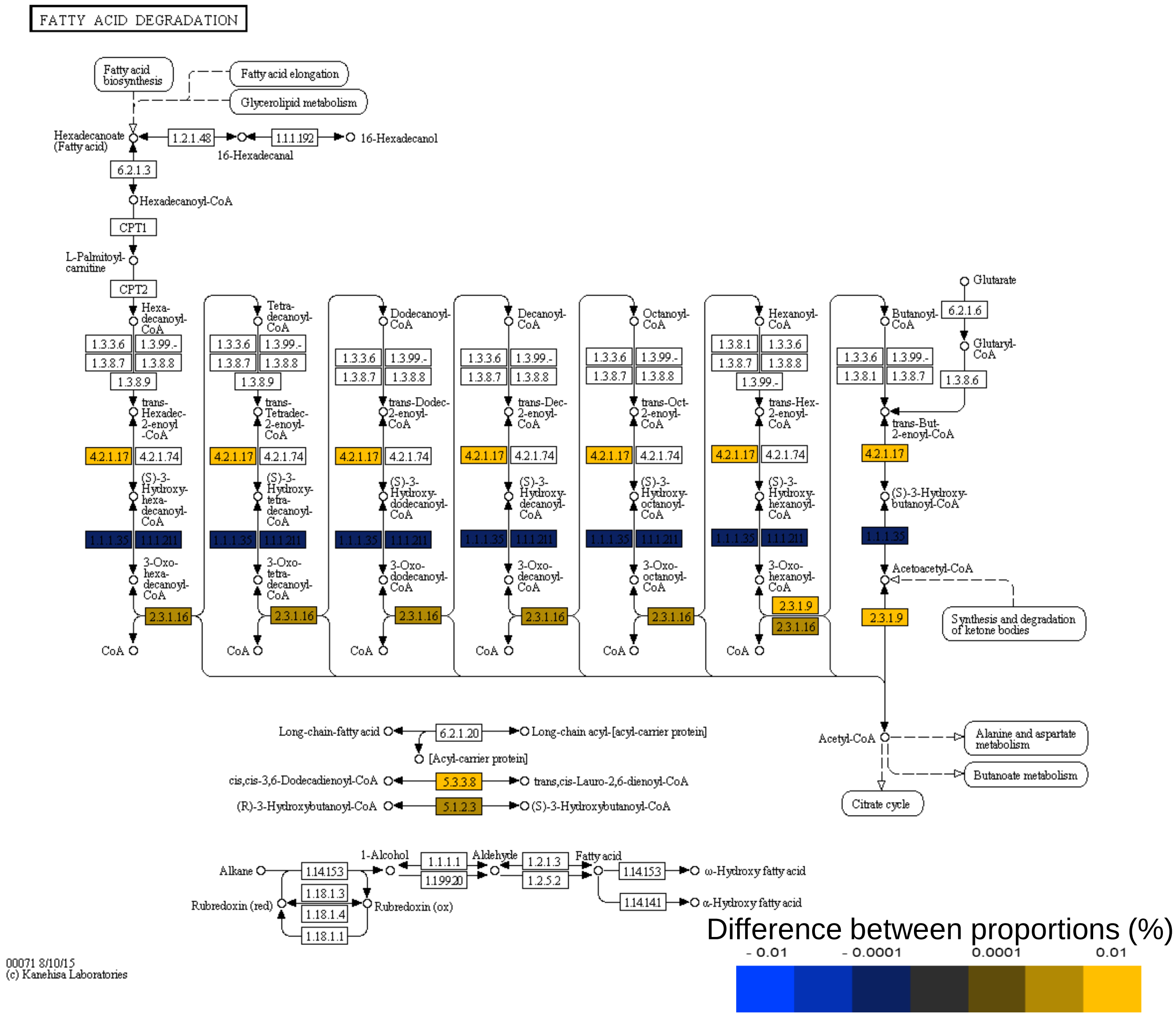
FATTY ACID DEGRADATION
Fatty acid biosynthesis
Fatty acid elongation
Glycerolipid metabolism
Hexadecanoate (Fatty acid)
1.2.1.48
16-Hexadecanal
1.1.1.192
16-Hexadecanol
6.2.1.3
Hexadecanoyl-CoA
CPT1
L-Palmitoyl-carnitine
CPT2
Glutarate
Hexa-decanoyl-CoA
Tetra-decanoyl-CoA
Dodecanoyl-CoA
Decanoyl-CoA
Octanoyl-CoA
Hexanoyl-CoA
Butanoyl-CoA
6.2.1.6
Glutaryl-CoA
1.3.3.6
1.3.99.-
1.3.8.7
1.3.8.8
1.3.8.9
1.3.8.1
1.3.8.6
trans-Hexadec-2-enoyl-CoA
trans-Tetradec-2-enoyl-CoA
trans-Dodec-2-enoyl-CoA
trans-Dec-2-enoyl-CoA
trans-Oct-2-enoyl-CoA
trans-Hex-2-enoyl-CoA
trans-But-2-enoyl-CoA
4.2.1.17
4.2.1.74
(S)-3-Hydroxy-hexa-decanoyl-CoA
(S)-3-Hydroxy-tetra-decanoyl-CoA
(S)-3-Hydroxy-dodecanoyl-CoA
(S)-3-Hydroxy-decanoyl-CoA
(S)-3-Hydroxy-octanoyl-CoA
(S)-3-Hydroxy-hexanoyl-CoA
(S)-3-Hydroxy-butanoyl-CoA
1.1.1.35
1.1.1.211
3-Oxo-hexa-decanoyl-CoA
3-Oxo-tetra-decanoyl-CoA
3-Oxo-dodecanoyl-CoA
3-Oxo-decanoyl-CoA
3-Oxo-octanoyl-CoA
3-Oxo-hexanoyl-CoA
Acetoacetyl-CoA
2.3.1.16
2.3.1.9
Synthesis and degradation of ketone bodies
CoA
Long-chain-fatty acid
6.2.1.20
Long-chain acyl-[acyl-carrier protein]
[Acyl-carrier protein]
Acetyl-CoA
Alanine and aspartate metabolism
cis,cis-3,6-Dodecadienoyl-CoA
5.3.3.8
trans,cis-Lauro-2,6-dienoyl-CoA
Butanoate metabolism
(R)-3-Hydroxybutanoyl-CoA
5.1.2.3
(S)-3-Hydroxybutanoyl-CoA
Citrate cycle
1-Alcohol
Aldehyde
Fatty acid
Alkane
1.14.15.3
1.1.1.1
1.2.1.3
1.14.15.3
ω-Hydroxy fatty acid
1.1.99.20
1.2.5.2
1.14.14.1
α-Hydroxy fatty acid
1.18.1.3
Rubredoxin (red)
Rubredoxin (ox)
1.18.1.4
1.18.1.1
00071 8/10/15
(c) Kanehisa Laboratories
Difference between proportions (%)
- 0.01
- 0.0001
0.0001
0.01

SUPPLEMENTARY FIG. S4. Taxa comparison of representative fecal bacterial communities between 16S rRNA gene and shotgun metagenomic sequencing analyses. The largest and the smallest sea cucumbers were compared at phylum level. We used reference database Greengenes and GenBank for 16S rRNA gene based analysis and metagenomic analysis, respectively.

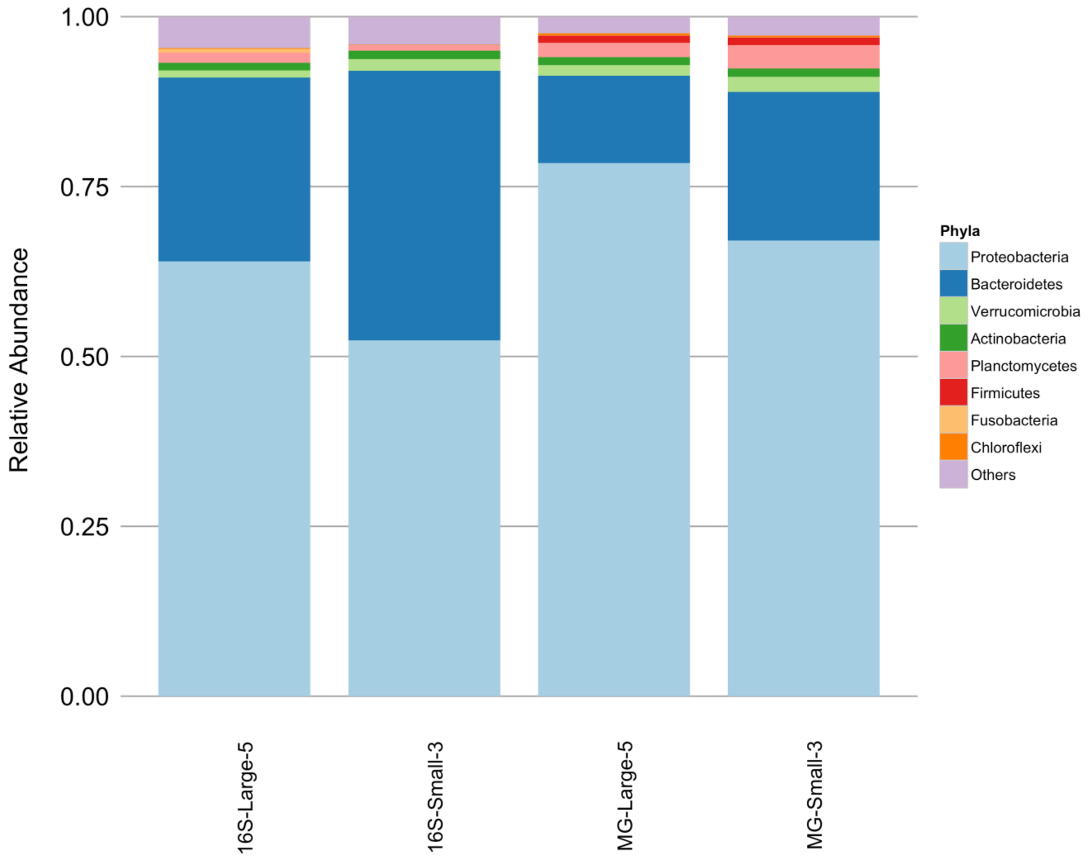

Supplement: Supplementary Information [file srep21631-s1.pdf]
